# Minimal interplay between explicit knowledge, dynamics of learning and temporal expectations in different, complex uni- and multisensory contexts

**DOI:** 10.3758/s13414-021-02313-1

**Published:** 2021-05-11

**Authors:** Felix Ball, Inga Spuerck, Toemme Noesselt

**Affiliations:** 1 Department of Biological Psychology, Faculty of Natural Science, Otto-von-Guericke-University Magdeburg, PO Box 4120, 39106 Magdeburg, Germany; 2 Center for Behavioral Brain Sciences, Otto-von-Guericke-University Magdeburg, Magdeburg, Germany

**Keywords:** Temporal expectations, Explicit knowledge, Statistical learning, Multisensory, Audio-visual, Reversal learning

## Abstract

While temporal expectations (TE) generally improve reactions to temporally predictable events, it remains unknown how the learning of temporal regularities (one time point more likely than another time point) and explicit knowledge about temporal regularities contribute to performance improvements; and whether any contributions generalise across modalities. Here, participants discriminated the frequency of diverging auditory, visual or audio-visual targets embedded in auditory, visual or audio-visual distractor sequences. Temporal regularities were manipulated run-wise (early vs. late target within sequence). Behavioural performance (accuracy, RT) plus measures from a computational learning model all suggest that learning of temporal regularities occurred but did not generalise across modalities, and that dynamics of learning (size of TE effect across runs) and explicit knowledge have little to no effect on the strength of TE. Remarkably, explicit knowledge affects performance—if at all—in a context-dependent manner: Only under complex task regimes (here, unknown target modality) might it partially help to resolve response conflict while it is lowering performance in less complex environments.

**Supplementary Information:**

The online version contains supplementary material available at 10.3758/s13414-021-02313-1.

## Introduction

Gathering temporal information is an essential aspect of our life. Every day, we use temporal information to determine when it is most likely we will catch the bus, or, in sports, we estimate when and where a ball has to be kicked, hit, or caught. Temporal regularities can be extracted from our surrounding, for example, by means of statistical learning (Hannula & Greene, 2012; Henke, 2010; Turk-Browne et al., 2009; Turk-Browne et al., 2010) and perceptual learning (Seitz, 2017; Seitz & Watanabe, 2009). The learning of temporal regularities typically results in temporal expectations (TE), expectations for specific moments in time (irrespective of target’s identity) that can improve performance (TE effect: faster and more accurate responses for targets expected in time). Computations resulting in TEs have been studied in various different experimental paradigms and different unisensory contexts (for review, see Nobre & Rohenkohl, 2014). However, most studies so far do not directly address the generalisability of TE effects, the related underlying learning mechanisms, as well as the impact of participants’ potential knowledge^1^ about temporal regularities on performance across different sensory systems. (Please note that we included a brief description of these relevant topics as well as a list of abbreviations in the Supplement to help readers unfamiliar with the presented concepts.)

Most previous studies on TE (for review, see Nobre & Rohenkohl, 2014) restricted tests to only one specific context (mostly unisensory visual events) and only one performance measure (mostly response times), which limited possible conclusions one can derive from the results. In particular, a focus on response times (RTs) makes it difficult to distinguish response preparation effects from TE-induced perceptual facilitations, with the latter having sometimes been reported in studies using accuracy measures for the auditory and visual modalities (Ball, Fuehrmann, et al., 2018; Ball, Michels, et al., 2018; Cravo et al., 2013; Rohenkohl et al., 2012). Also, changes in RTs might sometimes be due to shifts in decision criteria rather than response preparation and perceptual processing (Ball et al., 2020), an issue typically not addressed in previous RT-focussed studies. In addition, focussing on one modality comes at the cost that the successful manipulation of TE in laboratory settings does not always extend to real-life situations, which often comprise multisensory information. Further, it has been shown that multisensory stimulation often results in performance improvements relative to unisensory stimulation, for example, by means of stimulus interactions (called multisensory interplay/interaction; see, e.g., Alais & Burr, 2004; Ball, Fuehrmann, et al., 2018; Ball, Michels, et al., 2018; Driver & Noesselt, 2008; Noesselt et al., 2007; Noesselt et al., 2010; Parise et al., 2012; Starke et al., 2020; Werner & Noppeney, 2010) and that different sensory systems (e.g., auditory vs. visual) have different temporal properties (Burr et al., 2009; Fendrich & Corballis, 2001; Recanzone, 2003; Repp & Penel, 2002). Hence, the chosen stimulus modality potentially determines the presence or absence as well as the size of TE effects. Additionally, unisensory designs do not unequivocally support conclusions about potential cross-modal differences in TE. For instance, even if experiments with unisensory auditory and visual stimulations are compared, any difference between experiments might be simply driven by differences in groups or differences in experimental design (including differences in task difficulty based on, e.g., nonmatched stimulus intensities across experiments). Thus, within-subject designs, simultaneously manipulating TE in different sensory contexts, can help to minimize these confounds and to test for generalisability of TE across modalities.

Our previous work (Ball, Fuehrmann, et al., 2018; Ball, Michels, et al., 2018) established that TE can be observed for different modalities (auditory, visual, audio-visual) and improve perceptual sensitivity and response times for targets presented at expected moments in time. However, our results also suggest that the size of TE effects was enhanced and more robust in the multisensory as compared with the unisensory conditions. These conclusions derived from observed differences in average performance scores and led us to put forward the assumption that information about temporal regularities (during the learning process) is not generalised and thus might not be transferred across modalities. However, analyses of average scores simply ignore the fact that learning of certain features (here, temporal regularities) is a highly dynamic process. For instance, data can be largely dependent or independent on a trial-by-trial level, irrespective of presence or absence in average difference scores.^2^ To capture the dynamics of learning processes, the use of state-spaced learning models (Smith et al., 2004; Smith & Brown, 2003) has been proposed. These models estimate a learning curve based on single-trial accuracy data which can be used to determine not only the strength but also the speed of learning (defined by the identification of the first learning trial; Hargreaves et al., 2012; Smith et al., 2004). This type of modelling has been successfully applied to experiments with memory-association tasks, visuo-motor associative learning tasks, location-scene association and T-maze tasks (Clarke et al., 2018; Hargreaves et al., 2012; Smith et al., 2004; Wirth et al., 2003). However, to our knowledge the modelling of learning curves has not been applied to learning of temporal regularities so far, although it would allow for testing critical concepts about how temporal information is processed over time and whether temporal information is generalised across sensory systems. For instance, in multisensory paradigms (Ball, Fuehrmann, et al., 2018; Ball, Michels, et al., 2018) information about temporal regularities might either be transferred between modalities or learned independently for each modality. Hence, depending on whether the data are best described by a single learning curve (information transfer) or modality-specific learning curves (individual regularity learning) one could infer which learning form is the most likely for a given dataset/task. Further, experiments are mostly subdivided in runs (sequence of trials) with short breaks in-between. Hence, learning might be run-dependent (i.e., would be reset during runs, resulting in multiple learning curves) or there might be information transfer between runs (leading to one learning curve)^3^. Additionally, learning curves can be utilized to identify the onset of learning and whether this onset differs across experimental conditions and tasks (Clarke et al., 2018; Hargreaves et al., 2012; Smith et al., 2004; Wirth et al., 2003).

Turning to the potential effects of explicit knowledge of temporal information, differences between implicit and explicit knowledge about temporal regularities are often studied by comparing rhythm with cueing paradigms. In rhythm paradigms, a stimulus sequence is presented, either with a constant (expected) or varying delay (unexpected) between stimuli. In cueing paradigms, the cue determines with a certain probability (e.g., 80%) whether the target appears after a short or long cue-target delay period. The processing of rhythms is usually assumed to be under bottom-up (implicit TE) control (de la Rosa et al., 2012; Rohenkohl et al., 2011), while the processing and use of temporal cues is usually assumed to be under top-down (explicit TE) control (Coull & Nobre, 2008). This notion is in close resemblance to research on exogenous vs. endogenous orientation of spatial attention (Giordano et al., 2009; Kurtz et al., 2017; Müller & Rabbitt, 1989; Warner et al., 1990). Accordingly, several studies—independent of the applied paradigm—reported distinctions between ‘implicit’ and ‘explicit’ TEs on the behavioural and neural level (Capizzi et al., 2012; Correa et al., 2014; Coull et al., 2000; Coull & Nobre, 2008; Mento et al., 2013). Importantly, most previous studies did not directly assess participants’ explicit knowledge of the temporal manipulation. This leaves the possibility that even under supposedly implicit experimental task regimes, participants might have gained knowledge about the underlying temporal regularities which they may have been able to utilize to solve the particular task (Taylor & Ivry, 2013) rendering the assumed implicit task, a partially or fully explicit task. More importantly, others used different tasks and/or paradigms (e.g., cueing vs. rhythm tasks) for the comparison of implicit and explicit TE. However, when assessing differences of knowledge types not within but across experiments, any reported differences between implicit and explicit knowledge could actually be driven by differences in stimulation protocols across experiments; this includes the use of different stimulus material (e.g., same task but visual vs. audio-visual presentations; see Ball et al., 2020) or the comparisons of completely different experimental tasks and paradigms (e.g., entrainment [rhythm] vs. cueing experiments; see Correa et al., 2014). Thus, studies are needed that compare the effects of explicit knowledge within the same paradigm.

Previous non-TE-focussed studies investigating the influence of explicit knowledge—within the same paradigm—have reported divergent effects: While very few have observed an increase in performance (as measured by accuracies or response times), others have found no or even detrimental effects, or changes in confidence rather than performance (Batterink et al., 2015; Fairhurst et al., 2018; Green & Flowers, 2003; Mancini et al., 2016; Preston & Gabrieli, 2008; Sanchez & Reber, 2013; Van den Bussche et al., 2013). Recently, we were able to demonstrate that visual TE effects were not enhanced by explicit temporal knowledge in a simple visual TE task (Ball et al., 2020). Here, participants had to discriminate a target stimulus after an expected or unexpected cue–target delay (with the cue and delay being uninformative of target’s identity). The task was simple in the sense that targets were easily identifiable (cue was only followed by target display), accuracy was almost at ceiling (response times were mainly affected), and explicitly provided trial-specific temporal information was (at least in one explicit group) 100% valid (on each trial participants were informed about the upcoming cue–target delay). Together with reports from other fields, our previous findings suggest that explicit knowledge—when tested in the same experimental context—might not as readily affect performance compared with implicit statistical learning. However, it has yet to be tested whether the absence of explicit knowledge effects on TE is generalisable across the time course of the experiment (i.e., the learning process), different sensory contexts as well as different, more complex experimental paradigms.^4^ Most importantly, explicit knowledge might have important implications for the transfer of temporal information between modalities. Recognizing, for example, in one modality that targets appear at certain temporal position might motivate participants to attend the same temporal position in other modalities. Thus, explicit knowledge might ease the transfer of temporal information between modalities and thus, might also change the learning pattern.

In this study, we therefore tested the influence of continuous learning and explicit knowledge about task structure on performance and TEs—within the same paradigm—but under more complex task regimes (multisensory task with distractor stimuli). Here, we present a large-scale data set (*n* = 200) based on four different complex multisensory experimental designs in which we presented sequences of unisensory (auditory [A] or visual [V]) or multisensory (audio-visual [AV] stimuli with synchronous onsets) stimuli (all designs derived from Jaramillo & Zador, 2011; for a rationale of using this specific design, see Footnote 4). In Designs 1 and 2 (“easy” task), modality-specific uncertainty was low, meaning that with sequence onset, participants knew target’s modality (A sequence with A target, V sequence with V target, AV sequence with AV target). In Designs 3 and 4 (“difficult” task), modality-specific uncertainty was high—that is, with sequence onset, participants did not know target’s modality (AV sequence with A target, AV sequence with V target, AV sequence with AV target). Target stimuli (A, V, or redundant AV) appeared with a certain probability early or late within the stimulus sequence (11 stimuli presented successively, with one target embedded among 10 distractors). Likelihood of target occurrence within the sequence (at third or ninth position) was manipulated run-wise, with runs in which early or late targets were more likely.

Based on our previous reports (Ball, Fuehrmann, et al., 2018; Ball, Michels, et al., 2018), which only took into account average performance scores, we hypothesize that performance should be best explained by individual, modality-specific learning curves (modality-specific temporal regularity learning). Given that the TE effects were overall more robust in the audio-visual condition, we expected earlier learning trials (i.e., learning curve exceeding chance level) for the audio-visual compared with the unisensory target trials. Additionally, given learning effects—such as in contextual cueing experiments—one might expect that with repeated exposure, TE effects might be more pronounced. However, if information is transferred between expected and unexpected runs, TE effects might be rather stable across the experiment, as the TE effect solely depends on relearning the new target position in each run. Based on our previous publication and participants’ reports, we expected that explicit knowledge might be ineffective to modulate performance and the strength of TE. However, if explicit knowledge eases the transfer of information across modalities, we should find that the learning model best describing the data differs between participants with explicit and implicit temporal knowledge (one learning curve for all modalities vs. modality-specific learning curves). Furthermore, explicit knowledge might be associated with earlier learning onsets. Finally, if explicit knowledge modulates behaviour, we should find larger TE effects for participants with explicit knowledge, especially under complex experimental manipulations, which can make extraction of temporal regularities more difficult.

## Methods

Here, we addressed the novel research questions whether and how explicit knowledge and learning of temporal regularities affect the strength of TEs across different sensory contexts, and we focused on estimates derived from a learning model. Note that parts of the data set used here (Exps. 1–4, *n* = 120) were used in previous publications (Ball, Fuehrmann, et al., 2018; Ball, Michels, et al., 2018). However, here we extended (*n* = 80) the previous data set with data based on the same experimental designs, but belonging to so-far unpublished data sets. Thus, besides adding novel research questions and analyses, almost half of the data sets used here have not been analysed previously. In turn, these data sets help to increase the robustness of statistical estimations and to inform about the robustness of our previous results as well as test whether effects generalise to new populations of participants.

### Participants

In all experiments, participants were tested after giving signed informed consent. Volunteers who reported any neurological or psychiatric disorders or reduced and uncorrected visual acuity were excluded from the study.

Here, we used the same exclusion criteria as in our previous reports (Ball, Fuehrmann, et al., 2018; Ball, Michels, et al., 2018): Participants were excluded if they expressed a severe response bias (one response option used in more than 65% of all trials) and/or performance was well below chance level in one or more conditions (accuracy below 25%). This study was approved by the local ethics committee of the Otto-von-Guericke-University Magdeburg. In all experiments (for demographical data, see Table 1), we used an independent sample of naïve participants, except for Experiments 6_1 and 6_2 (experiment with two sessions per participant). Note that for Experiments 6_1 and 6_2, each participant could have been excluded (based on our exclusion criteria) for Exp. 6_1 but not Exp. 6_2 and vice versa (hence, samples were only almost identical).

**Table 1:** Demographical data of each of the experiments

Experiment	Design	Mean age ± *SD*	Women	Left-handed	Total
Exp. 1	1	24.5 ± 2.7	13	2	30
Exp. 2	2	23.1 ± 3.4	18	0	30
Exp. 3	3	24.3 ± 3.6	21	4	30
Exp. 4	4	23.9 ± 3.7	22	2	30
Exp. 5	2	23.6 ± 2.7	15	3	21
Exp. 6_1	2	23.3 ± 4.7	22	1	30
Exp. 6_2	4	22.9 ± 4.3	22	1	29

*Note*. See Fig. 1 for a description of all four experimental designs

### Apparatus

The experiments were programmed using the Psychophysics Toolbox (Brainard, 1997) and MATLAB 2012b (The MathWorks Inc., Natick, MA). Stimuli were presented on an LCD screen (22-in., 120 Hz, SAMSUNG 2233RZ) with optimal timing and luminance accuracy for vision researches (Wang & Nikolić, 2011). Resolution was set to 1,650 × 1,080 pixels, and the refresh rate to 60 Hz. Participants were seated in front of the monitor at a distance of 102 cm (eyes to fixation point). Responses were collected with a wireless mouse (Logitech M325). Accurate timing of stimuli (≤1 ms) was confirmed with a BioSemi Active-Two EEG amplifier system connected with a microphone and photodiode.

### Stimuli

Unisensory or multisensory stimulus sequences (pure tones, circles filled with chequerboards, or a combination of both) were presented on each trial. Chequerboards subtended 3.07° visual angle, and were presented above the fixation cross (centre to centre distance of 2.31°). Sounds were presented from one speaker placed on top of the screen (Designs 1 and 3) at a distance of 7.06° from fixation, 4.76° from chequerboard’s centre, and 3.22° from chequerboard’s edge or via headphones (Sennheiser HD 650; Designs 2 and 4). The speaker was vertically aligned with the centre of the chequerboard stimulus. Chequerboards were presented on a dark-grey background (RGB: 25.5). The fixation cross (white) was presented 2.9° above the screen’s centre.

Chequerboards and pure sounds were used as targets and distractors. The distractor frequencies were jittered randomly between 4.6, 4.9, and 5.2 cycles per degree for chequerboards and between 2975, 3000, and 3025 Hz for sounds. Visual and auditory target frequencies were individually adjusted to a 75% accuracy level at the beginning of the experiment. Hence, targets—although the same type of stimulus (chequerboard/pure sound)—were either lower or higher in frequency compared with distractor frequencies. Furthermore, the intensities for both target and distractor chequerboards and sounds were varied randomly throughout the stimulus sequences. The nonwhite checkers were jittered between 63.75, 76.5, and 89.25 RGB (average grey value of 76.5 RGB). The sound intensities were jittered between 20%, 25%, and 30% of the maximum sound intensity (average of 25% = 52 dB[A]). The sound intensity in the experiments with headphones was adjusted to match the sound intensity used for speaker experiments.

### Procedure

**Fig. 1:**
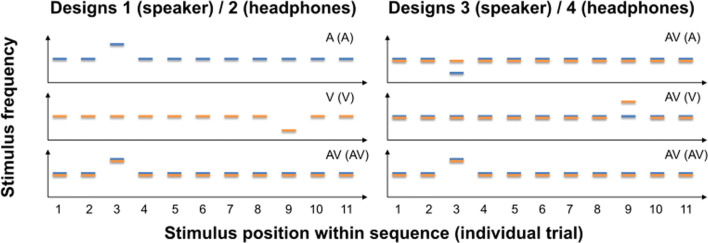
Schematic examples for stimulus sequences in Experimental Designs 1 to 4 (blue = auditory [A], orange = visual [V], blue + orange = audio-visual [AV]). (Left) Stimulus sequences used in Experimental Designs 1 and 2. Each bar represents one of 11 stimuli with a specific stimulus frequency. Bars above or below distractors (auditory distractor frequency: 2975–3025 Hz, visual distractor frequency: 4.6–5.2 cycles per degree) depict high or low frequency targets, respectively. Targets were only presented at Position 3 or 9. The modality of the sequence are depicted in the upper right corner of each graph, with the modality of the target in brackets (e.g., AV [AV] = audio-visual sequence with audio-visual target). (Right) In Experimental Designs 3 and 4, we only presented audio-visual sequences, but targets could be either unimodal (A or V) or bimodal (AV). Whenever a target was bimodal, both auditory and visual stimuli were *always* either higher or lower in frequency compared with distractors (redundant target). Note that we only show examples but not all combinations of factors early/late target, higher/lower frequency target and target modality (A, V, AV) which were completely counterbalanced in all experiments. (Colour figure online)

Participants were seated in a dark, sound-attenuated chamber. For each trial, a sequence consisting of 11 stimuli was presented (see Fig. 1 for example sequences). Stimulus duration was 100 ms, and stimuli were separated by a 100-ms gap. All stimuli within a sequence were either auditory, visual, or combined auditory and visual stimuli (synchronous presentation). Target stimulus pairs on multisensory trials were always redundant—that is, targets of both modalities had congruently either a lower or higher frequency than distractors. For each trial, we presented one target stimulus or target stimulus pair (audio-visual sequences) at the third (onset at 400 ms, early target) or ninth position (onset at 1,600 ms, late target) of the sequence.^5^ Participants were instructed to maintain fixation throughout the experiment and were told that a target was present in each trial. They were required to discriminate the frequency (low or high) of the target as quickly and accurately as possible using a two-alternative forced-choice (2AFC) procedure. Participants held the mouse with both hands, while placing their left/right thumbs on the left/right mouse buttons, respectively. Each button was used for one of the two response options (key bindings were counterbalanced across participants). The response recording started with the onset of the first stimulus of the sequence and ended 1,500 ms after sequence’s offset. Only the first button press was recorded. The end of the response period was then followed by a 200–400-ms intertrial-interval. In case no button was pressed, the trial was repeated after each run’s quarter (mean of repeated trials across participants: 0.7% ± 1.4% *SD*).

Each experiment contained three sessions: an initial training session to familiarise participants with the task, a threshold determination session, and the main experimental session. During *‘training’* (24 trials) and *‘threshold determination’* runs (144 trials), we presented unisensory sequences only (auditory or visual). Low and high frequency, early and late occurring, and auditory and visual targets were balanced in these runs. Prior to the experimental sessions, we always conducted two threshold determination runs. After threshold acquisition, visual and auditory stimuli were individually adjusted to 75% accuracy for all the aforementioned conditions. Each ‘*main experimental session’* was separated into six runs (168 trials per run, i.e., 1,008 trials total), and we presented all stimulus types (unisensory auditory and visual and multisensory stimuli) and modulated TE by presenting different numbers of early and late targets within runs. An 86% likelihood of early target occurrence (always at the third position) and a 14% likelihood of late targets (ninth position) within the stimulus sequence was used for “expect early” runs. In “expect late” runs, early target occurrence was reduced to 43%. We chose this procedure instead of a complete reversal of probabilities in order to obtain a robust estimate of the performance in unexpected early trials, as early trials were at the focus of our analysis (see below). Expected and unexpected runs (three runs each) alternated throughout the experiment, and the type of the first run was counterbalanced across participants. Importantly, all participants were naïve with regard to the changing likelihoods of target position across runs at the beginning of the experiments. Within each run, the number of trials was balanced for sequence types, target types, and target frequencies. Additionally, the number of auditory, visual, and multisensory stimuli, early and late, and low and high targets was balanced across each quarter of runs.

Note that in this manuscript we differentiate between experiments and experimental designs as some of the experiments have the same underlying experimental design but data collection was based on a new population of participants. The overall procedure for each experiment (1 to 6_2; for individual design, see Table 1) was identical with the following exception: Modality-specific uncertainty was modulated by changing the type of stimulus sequences across experiments. In one experimental design (Designs 1 & 2), we presented either unisensory sequences with unisensory targets or multisensory sequences with multisensory redundant targets (low modality-specific uncertainty). During high modality-specific uncertainty (Designs 3 & 4), we presented only audio-visual sequences, BUT, targets were, as before, either unisensory (auditory or visual) with a concurrent distractor in the second modality or redundant multisensory targets (high modality-specific uncertainty). Thus, to perform the task, participants were forced to equally monitor both modalities on each trial to be able to detect the target. Furthermore, auditory stimuli were either delivered over speakers attached in close proximity to the visual stimulus (Designs 1 & 3; attend front only) or headphones (Designs 2 & 4; attend front [visual] and inner of the head [sound]) to further modulate attention.

#### Postexperimental assessment of explicit knowledge

To assess whether participants gained explicit knowledge about temporal structures, we conducted a postexperimental interview. Note that the classification of explicit and implicit knowledge based on verbal reports is a common procedure and has successfully been used in previous studies (Ball et al., 2020; Ewolds et al., 2017; Heuer & Schmidtke, 1996; Nissen & Bullemer, 1987). We asked the following questions to gain exhaustive insight into participants’ explicit state of knowledge about temporal regularities. The order of questions was kept constant. Note that early questions were open to avoid biasing, but later questions were more specific to encourage participants to report even vague explicit insights:

- Did you notice any regularities throughout the experiment?
- Was there something specific about the position of target appearance within the sequence?
- Could you please guess at which positions the target was presented?
- Was there a difference between odd and even runs?
- Was there a position pattern throughout the run, or was it random across trials?
- Could you please guess the difference between runs?

If participants were unsure about the meaning of ‘trial’ and ‘run’, we further elaborated on the meaning of these terms (trial = one sequence of 11 stimuli for which the frequency of a target had to be reported, run = all trials between two breaks). Only if the answers to all questions were negative—that is, participants reported the target to appear at all positions or at clearly incorrect positions (e.g., “Target was always presented at the sixth position”), participants were labelled as having only implicit knowledge. Whenever they gave a correct or at least partly correct answer (e.g., “Target was mostly presented early”), they were labelled as having explicit knowledge. We opted for including partly correct answers because exact stimulus positions were difficult/impossible to count given the stimulation frequency (Ball, Fuehrmann, et al., 2018; Ball, Michels, et al., 2018) and participants were at least aware that stimuli occurred mostly early in the sequence (i.e., not the middle or end).

### Analysis

In our previous report (Ball, Michels, et al., 2018), we used a criterion to remove response outliers and confirmed—by applying multiple criteria—that the removal of outliers did not affect the results. The present analyses required the same number of trials across participants and conditions to calculate the learning curves. To this end, responses faster than 150 ms were *not excluded* but rather labelled ‘incorrect’, as they are unlikely based on processing of perceptual input. Note that target’s frequency was unpredictable on a trial-by-trial basis (50% low, 50% high frequencies) and had to be classified (frequency judgement). Thus, responses prior to target presentation or before the target is sufficiently processed cannot be linked to effects of TEs, response preparation, or such like and simply represent a premature response. To confirm that this labelling does not change our previous results, we reran all our prior analysis (Ball, Michels, et al., 2018). The results (see Supplement 1) are virtually identical to the ones in our previous report. Thus, our previously presented effects appear to be rather robust to the choice of outlier treatment.

Furthermore, as in our previous publications and publications by other groups, we test for TE effects by focussing on early target trials; late targets are always expected and may thus not require temporal attention (see Ball, Fuehrmann, et al., 2018; Ball, Michels, et al., 2018, for a demonstration of the absence of late target TE effects for the present data set; see also Jaramillo & Zador, 2011; Lange & Röder, 2006; Lange et al., 2003; Mühlberg et al., 2014, for similar a approach).

#### Learning model selection

One part of our analyses was based on utilising an established learning model algorithm to analyse our data. In short, the algorithm estimates the learning process by fitting a learning curve to the single trial accuracy data. Here we used the logistic regression algorithm developed by Smith and colleagues (Smith et al., 2004; Smith & Brown, 2003). The algorithm (used in MATLAB 2017b; The MathWorks Inc., Natick, MA) takes into account the binary responses on each trial (correct trials: 1, incorrect trials: 0) and fits a learning curve to the data by using a state-space model of learning, in which a Bernoulli probability model describes behavioural task responses and a Gaussian state equation describes the unobservable learning state process (Kakade & Dayan, 2002; Kitagawa & Gersch, 1996; Smith et al., 2004; Wirth et al., 2003). Furthermore, the algorithm is based on an expectation maximization algorithm (Dempster et al., 1977) which computes the maximum likelihood estimate of the learning curve (including the respective confidence intervals).

While the individual learning curves can be used to assess, for example, when a participant has learned the regular temporal pattern (i.e., on Trial X), the learning model itself can be used to test different assumptions about whether and how information is transferred across modalities (AV, A, V) and runs. To identify which underlying process best describes the data, we tested three different models by computing the learning curves on different portions of the trials (see Table 2). For instance, if participants learn temporal regularities (i.e., target appears at Position 3) independently for each modality, we should observe that the best model is based on individual learning curves for each modality (here, Model 2; see Table 2). The best model was determined for each participant by calculating the Akaike information criterion (AIC) for each model, transforming the AIC into AIC weights and finally calculating the evidence ratio for each model (see Wagenmakers & Farrell, 2004, for mathematical equations). The evidence ratio indicates how likely each model is compared with the best model. Naturally, the best model has an evidence ratio of 1 (best model score divided by itself = 1). If the comparison model has a score of, for example, .5 or .33, it means that this model is 2 or 3 times less likely than the best model (in close resemblance to the Bayes factor). To foreshadow, Model 2 was on average as well as individually the best model across all experimental designs.

**Table 2:** Description of individual learning models and the related assumptions

Model	Learning curves	Assumption
M1	Learning curve for all early target trials	• Information generalised across modalities • Information transferred across runs
M2	Learning curve for each condition (3 curves total)	• Information NOT generalised across modalities • Information transferred across runs
M3	Learning curve for each condition and for each run (18 curves)	• Information NOT generalised across modalities • Information NOT transferred across runs

#### Data analyses

In addition to the learning curve analysis (see above), three different performance measures were analysed: mean percentage correct, mean response times, and the ‘learning trial’. The learning trial was specified as the first trial for which the lower confidence interval of the learning curve reliably exceeded chance level (here, .5 due to the 2AFC) and stayed above threshold till the end of the experiment (Smith et al., 2004). Learning trials were calculated based on the overall best learning model. For accuracies and RT, we calculated the average scores dependent on factors TE (expected, unexpected), modality (AV, A, V), and run (Runs 1 & 2, Runs 3 & 4, Runs 5 & 6) to test for effects of dynamics of learning (change of TE effect across runs) and modality on TE. Further, we added the factors spatial uncertainty (low, high) and knowledge (implicit, explicit). Note that “modality-specific uncertainty” could not function as a direct interaction term due to the partially crossed design. However, our previous reports revealed that only spatial uncertainty significantly influenced the interaction of modality and TE (Ball, Fuehrmann, et al., 2018; Ball, Michels, et al., 2018), while modality-specific uncertainty contributed the least to TE effects.

For analyses, we used MATLAB 2017b (the MathWorks Inc., Natick, MA), R (v. 4.0.0), and RStudio (v. 1.0.153). For statistical analyses, we used the ‘afex’ package (v. 0.27-2) in R, as data were partially crossed (for factor modality-specific uncertainty). To identify which combination of factors affects TE effects most, we used again a model comparison approach. To this end, we defined all possible interaction models of the factors modality, run, spatial uncertainty, and knowledge with factor TE as well as a pure intercept model (see Supplement 2 for overview). To account for the partially crossed data structure, each model also modelled random effects intercepts based on factors modality-specific uncertainty and participant. Hence, we used 16 random intercept models for comparison based on the AIC evidence ratio. For learning trials, we formed all interaction models based on factors modality, knowledge and spatial uncertainty (eight models) for comparison. After determining the best model for each performance measure, individual differences between conditions within the winner model were analysed by using *F*-values (calculated with the KR method) and post hoc *t* tests (calculated using the ‘emmeans’ package, v. 1.4.7, with asymptotic dF).

Learning curves were analysed in MATLAB by means of cluster permutation tests (for introduction, see Maris & Oostenveld, 2007) to identify potential differences between modalities as well as knowledge groups. In short, the test identifies consecutive trials with consistently significant results (e.g., Trials 4 to 15 = cluster), sums e.g., the *T *statistic across these trials and tests whether such result would also be observed if the data were random. To determine differences between conditions, we used a significance threshold of .05 to identify potential clusters in time (consecutive time points with significant result) and a final cluster-threshold of .004 (Bonferroni corrected to account for multiple testing; note that false-discovery-rate correction resulted in the same outcome). Below, we report the corrected cluster-threshold *p* values (p_cluster_). For the comparison of modalities, we used dependent-sample *t* tests, and for comparisons of knowledge groups, we used independent sample *t* tests to determine the maximum sum *t* value of each ‘original data’ cluster as well as the ‘random permutation’ clusters which are used to form the null hypothesis. Note that the cluster test procedure required that learning curves differences are tested separately for low and high modality-specific uncertainty. Hence, we also repeated our model selection approach for the abovementioned data scores (now split for modality-specific uncertainty) to compare it to the outcome of the cluster analyses.

## Results

### Results of postexperimental questionnaire

In 58 cases out of the 200 collected data sets, participants noticed any position regularity (Exp. 1: 12, Exp. 2: 9, Exp. 3: 10, Exp. 4: 6, Exp. 5: 6, Exp. 6_1: 7, Exp. 6_2: 8). In 15 cases, participants stated regularities immediately. All others only reported regularities after follow-up questioning. A total of 41 participants could identify the second or third position as target position, while the remaining stated that targets “occurred mostly early” or “mostly early and late”. Out of the 58 participants, 11 made their statements specifically for the auditory but not visual stimulus.

### Learning model selection results

**Fig. 2:**
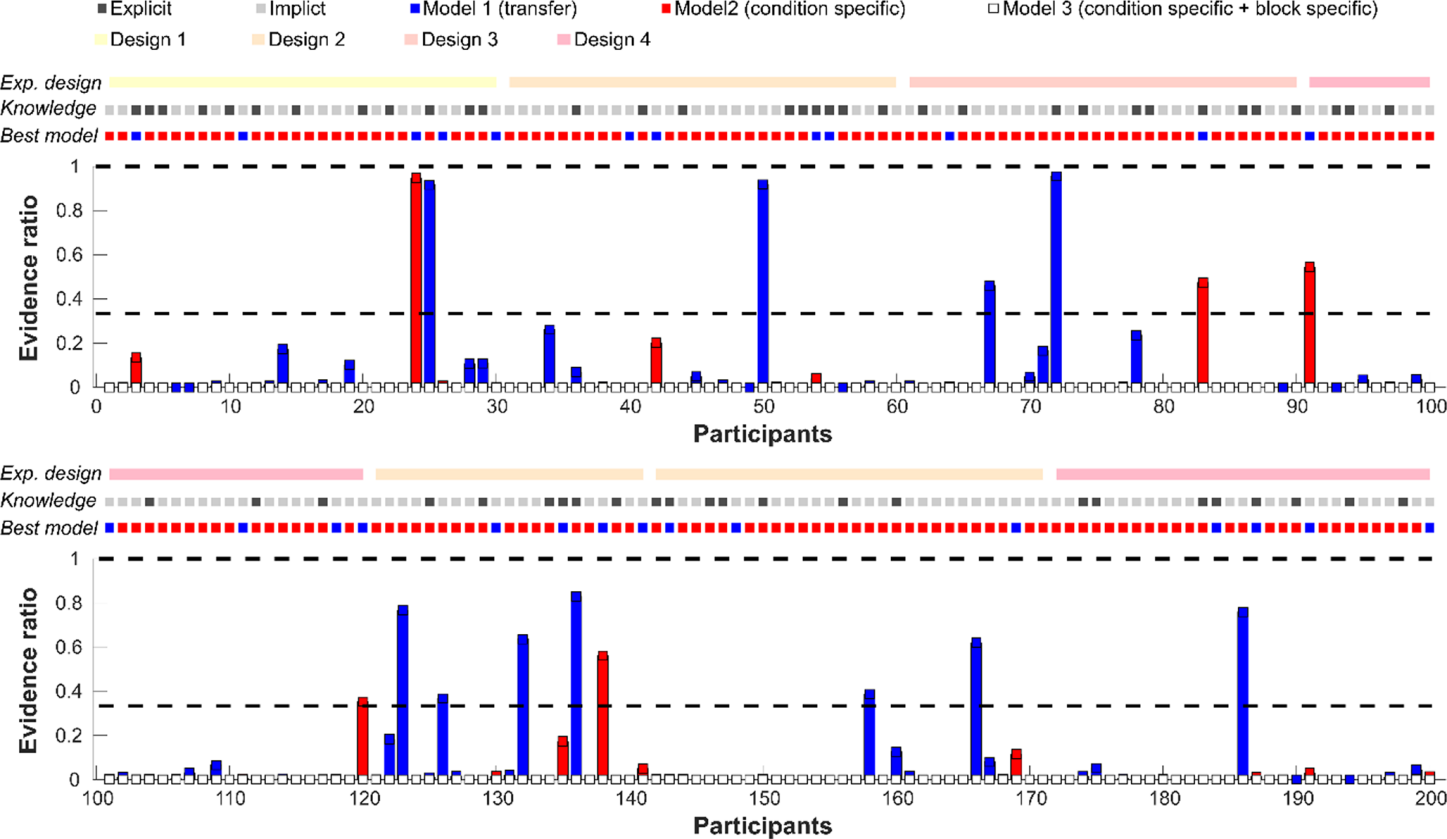
Evidence ratio for each learning model (based on estimated learning curves). Each number on the *x*-axis represents an individual participant. Top: Participants 1–100 (Exps. 1–4). Bottom: participants 101–200 (Exps. 4–6_2). The top dotted lines indicate the experimental design (Design 1: low spatial and low modality-specific uncertainty; Design 2: high spatial and low modality-specific uncertainty; Design 3: low spatial and high modality-specific uncertainty; Design 4: high spatial and high modality-specific uncertainty), participants’ knowledge (light grey = implicit, dark grey = explicit) and the individual best model (blue = Model 1 [single learning curve], red = Model 2 [modality-specific learning curves], white = Model 3 [modality-specific and TE specific learning curves]). Please note that Model 3 was never the best model. The bar graph below depicts individual evidence ratios for the second and third best models as compared with the best model. A score of .5, for example, implies that the second or third best model was 2 times less likely than the best model. Here, we highlighted the .33 score, as this value indicates that the individual model was 3 times less likely than the best model (in close resemblance to Bayes factor analyses). (Colour figure on line)

The learning model that best described the single-trial data across all participants (86.5% of participants) was Model 2 (see Table 3): Here, the data were modelled independently for each modality, while expected and unexpected trials were not further split up. Thus, the best model suggests that information about the most likely temporal position is not generalised across modalities, but that information about the temporal position (early targets) is carried over between runs. Note that this result was independent of explicit knowledge, experiments, and design types (see Table 3). A depiction of the model evidence ratios is displayed in Fig. 2.

**Table 3:** Subject-specific best-fitting model (Models 1–3) separated by participants’ temporal awareness

	Implicit group				Explicit group			
Experiment	*N*	Model 1	Model 2	Model 3	*N*	Model 1	Model 2	Model 3
Exp. 1	18	4	14	0	12	1	11	0
Exp. 2	21	2	19	0	9	2	7	0
Exp. 3	20	1	19	0	10	1	9	0
Exp. 4	24	5	19	0	6	0	6	0
Exp. 5	15	3	12	0	6	1	5	0
Exp. 6_1	23	2	21	0	7	1	6	0
Exp. 6_2	21	2	19	0	8	2	6	0
*Total*	*142*	*19*	*123*	*0*	*58*	*8*	*50*	*0*

*Note.* Rows: Number of participants are listed separately for each experiment as well as summarized across experiments (‘Total’). Columns: Number of participants’ are listed based on the best-fitting-model and participants’ awareness of temporal regularities (explicit and implicit groups) plus total explicit and implicit group size (***N)***. Model 1 (single learning curve), Model 2 (modality-specific learning curves), Model 3 (modality-specific and TE specific learning curves).

### Average performance scores analyses—Whole data set

**Fig. 3:**
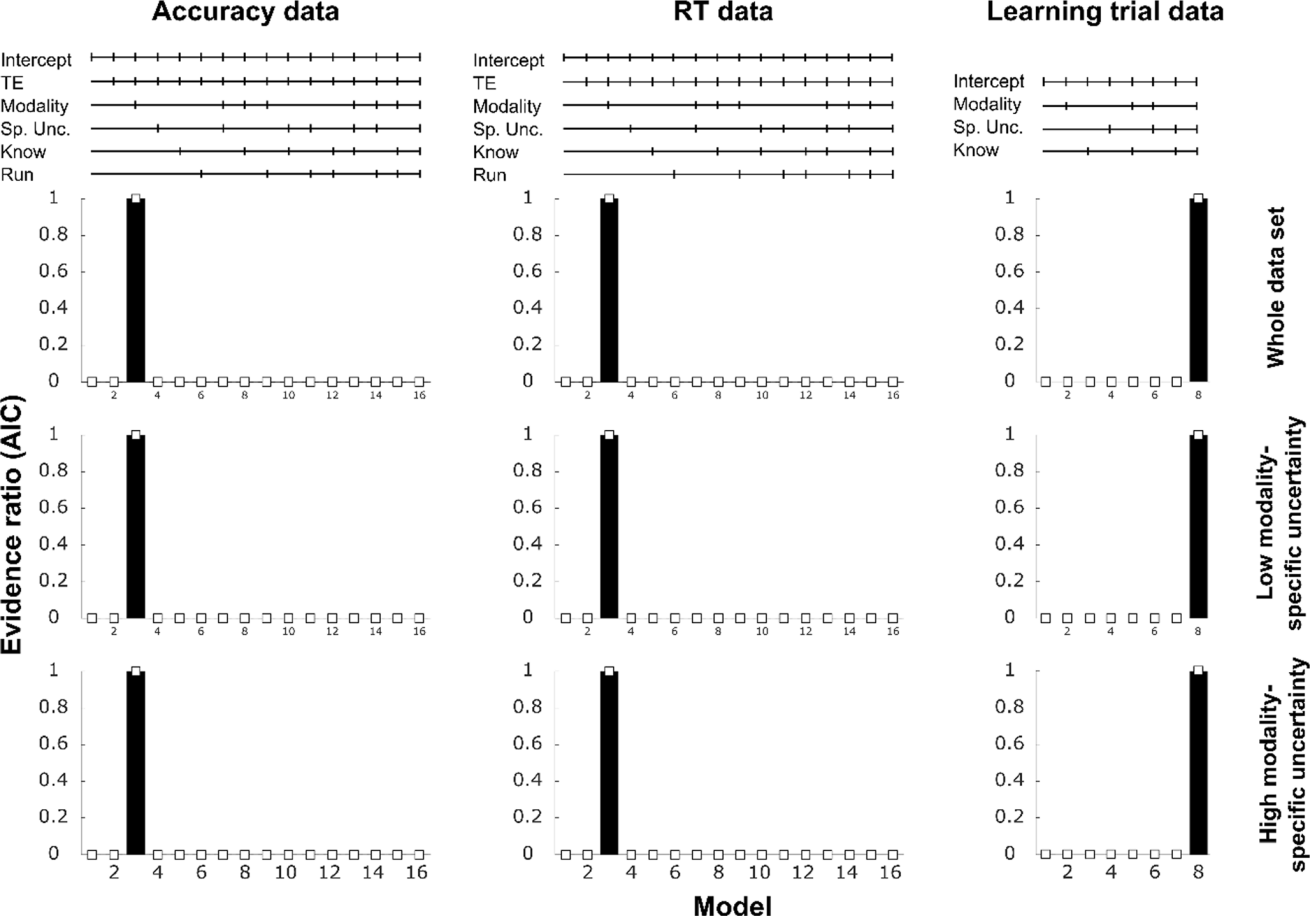
Comparison of mixed-models for each average performance score based on AIC evidence ratio. Accuracy (left column), RTs (middle column), and learning trial (right column). The top line graphs depict the factors that were included as interaction terms in the respective model (Sp. Unc. = spatial uncertainty; Know = knowledge group). Bar graphs below show the evidence ratios for analyses of the whole data set (top row) and the split into low and high modality-specific uncertainty data (middle and bottom row). Note that the best model has an evidence ratio of 1 (best model score divided by itself = 1), and that, for instance, a score of .333 implies that the comparison model was three times less likely than the best model. More importantly, the graph shows that all ‘non-best’ models showed evidence ratios close to zero (as indicated by the white square markers close to the *x*-axis), indicating that these models are highly unlikely as compared with the best model

The model selection, based on the AIC evidence ratio, suggested a clear winner model for each of the three performance measures. For both, the accuracy (Acc) and the RT data, the winning model consisted of the interaction of TE × Modality. For the learning trial data, the full model (Modality × Spatial Uncertainty × Knowledge) accounted best for the data at hand. Note that the best model for each performance measure was the same, independent of whether we analysed the whole data set or only the low or high modality-specific uncertainty data (see Fig. 3).

**Fig. 4:**
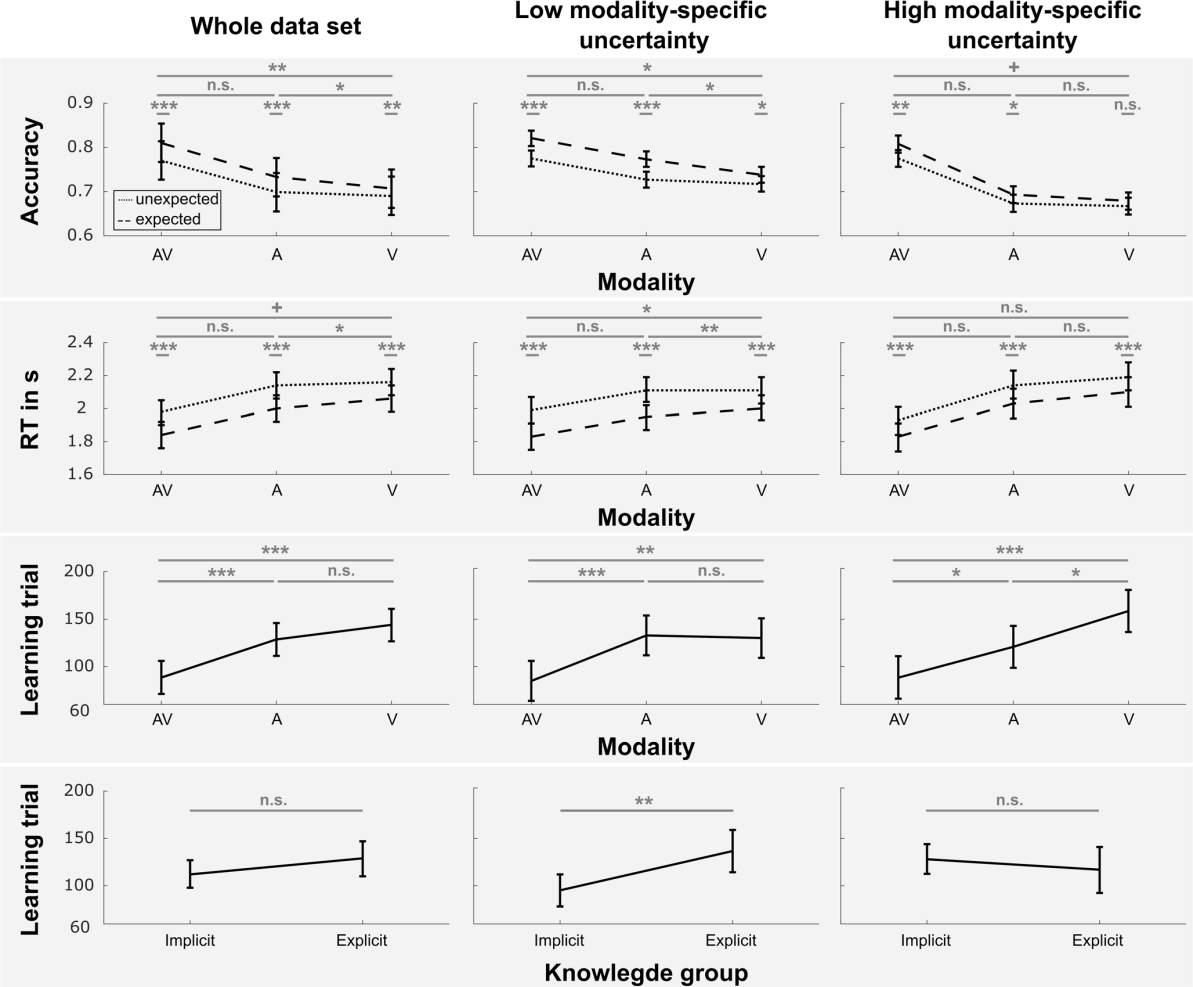
Group-mean scores (estimated marginal means) for accuracy (Row 1), RT (Row 2), and learning trial (Rows 3–4) effects. Averages and confidence intervals (error bars) are presented for analyses of the whole data set (left) as well as for the split into low (middle) and high modality-specific uncertainty data (right). Statistical comparisons (uncorrected) are labelled as follows: n.s. (*p* > .1), + (*p* > .05), * (*p* > .01), ** (*p* > .001), *** (*p* ≥ 0). Note that in the top two rows, data is split into expected (dashed line) and unexpected (dotted line) trials. Further, statistical results displayed in the top two rows are based on testing the difference between expected and unexpected trials as well as testing whether this difference was altered by the modality context. Rows 3–4 displays modality-specific and knowledge-specific effects of the learning trial

Following up on the individual differences in estimated marginal means, we found a significant main effect of TE, Acc: *F*(1, 3442.16) = 70.21, *p* < .001; RT: *F*(1, 3442.01) = 274.62, *p* < .001, a main effect of modality, Acc: *F*(2, 3442.16) = 241.56, *p* < .001; RT: *F*(2, 3442.01) = 264.63, *p* < .001, as well as an interaction of both factors, Acc: *F*(2, 3442.16) = 3.8, *p* = .022; RT: *F*(2, 3442.01) = 3.0, *p* = .05, for both the accuracy and RT data. Summarized, participants responded more often correctly and faster in the expected compared with the unexpected condition, more correctly in the AV compared with the A and V conditions, and the TE effect was enhanced in the AV and A conditions as compared with the V condition (see Fig. 4, top two rows).

For the learning trials, only the factor modality was significant, *F*(2, 422.99) = 16.41, *p* < .001, with earlier learning trials in the AV compared with the A and V conditions (see Fig. 4, Row 3). Although other factors contributed to explaining variance of the data, the effects themselves were nonsignificant (all *F*s < 2.5, *p*s > .115).

### Average performance scores analyses—Low modality-specific uncertainty data (known target modality)

The model selections for accuracy and RT data, based on low modality-specific uncertainty only, were in line with the model selection for the whole data set (see Fig. 4, middle). The best model in both cases was Model 2 (see Fig. 3, Row 2), which was based on the interaction of TE and modality. Again, we found significant main effects of TE, Acc: *F*(1, 1882) = 64.06, *p* < .001; RT: *F*(1, 1882) = 229.97, *p* < .001, and modality, Acc: *F*(2, 1882) = 77.67, *p* < .001; RT: *F*(2, 1882) = 90.74, *p* < .001, as well as the interaction of both terms, Acc: *F*(2, 1882) = 3.23, *p* = .04; RT: *F*(2, 1882) = 4.34, *p* = .013, with essentially the same results pattern as already described for the whole data set. Performance was elevated in the expected condition, in the audio-visual condition, and TE effects were enhanced in the AV and A compared with the V condition (see Fig. 4, middle).

For the learning trials, the results were also virtually identical, with a similar effect of modality on the learning trial, modality: *F*(2,214) = 7.64, *p* < .001 (see Fig. 4, Row 3). However, here, also, the main effect of knowledge was significant, *F*(1, 107) = 8.4, *p* = .005 (all other effects *F* < 2.38, *p* > .095), indicating earlier learning trials for the implicit compared with the explicit group (see Fig. 4, Row 4).

**Fig. 5:**
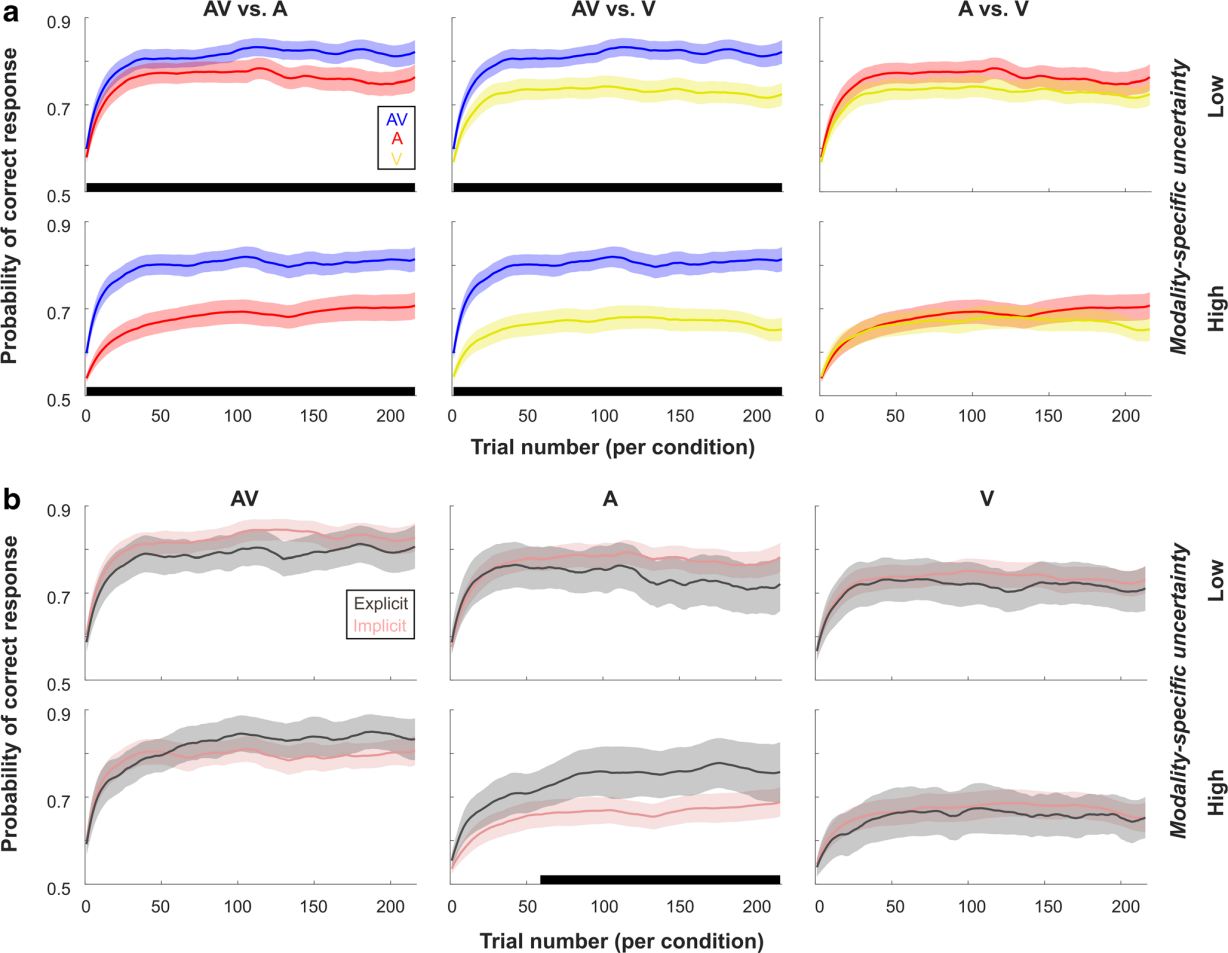
Learning curve data comparison for modalities (**a**) and knowledge groups (**b**). Time ranges of significant clusters (consecutive significant differences across trials between conditions) are highlighted with black bars. Results are presented for low (top rows) and high (bottom rows) modality-specific uncertainty. Colours used for conditions: AV–blue, A–red, V–yellow, Explicit group–dark grey, Implicit group–light red. (Colour figure online)

The analyses of learning curves mirrored the results for the accuracy data (note that we did not split data for low and high spatial uncertainty, based on the outcome of the mixed model comparison procedure). First of all, there was a clear-cut modality effect: performance was increased in the AV compared with the A (1 cluster with p_Cluster_ = 0) and V conditions (1 cluster with p_Cluster_ = 0). Although, cluster time ranges have to be interpreted cautiously (Sassenhagen & Draschkow, 2019), the cluster results suggest that conditions differed across the whole time range of the experiment. Thus, the constantly higher probability of responding correctly was clearly driven by multisensory stimulation, but less driven by learning (especially for the comparison with the visual modality). No differences were found between the A and V conditions (all p_Cluster_ > .252). Similarly, we found no significant differences (all p_Cluster_ > .468) for the comparison of participants with explicit and implicit knowledge. More importantly, even if the groups would differ, performance would be lower in the explicit knowledge group—the opposite of the effect one would expect for the influence of explicit knowledge on behaviour (see Fig. 5). Additionally, the results in Fig. 5 strongly suggest that after the initial onset, performance varied only minimally across time.

### Average performance scores analyses—High modality-specific uncertainty data (unknown target modality)

Again, Model 2 was the best-fitting model for the accuracy and RT data, and Model 8 was the best-fitting model for the learning trials (see Fig. 3, Row 3). Note that there were two differences to the abovementioned results. While the main effects of TE and modality were still significant (all *p*s < .001), the interactions of TE × Modality—for accuracies as well as response times—did not reach significance under high modality-specific uncertainty, Acc: *F*(2, 1508) = 1.39, *p* = .25; RT: *F*(2,1508) = .22, *p* = .799.

Additionally, the modality effect for the learning trials was not restricted to the AV condition anymore: learning trials were not only faster for the AV compared with both unisensory conditions but also for the A compared with the V condition, *F*(2, 170) = 11.26, *p* < .001 (see Fig. 4, Row 3). All remaining effects related to the learning trials were nonsignificant (*F*s < 1.66, *p*s > .193).

Similar to results from the low uncertainty condition, analyses of the learning curves showed again a significant modality effect (see Fig. 5), with performance being higher in the AV condition compared with both unisensory conditions (p_Cluster_ = 0). There were again no significant differences between the A and V condition (p_Cluster_ > .756). However, for the comparison of participants with explicit and implicit knowledge, there was a significant difference in the auditory condition (p_Cluster_ = .046). The cluster was spanning the mid to end trials of the experiment, with higher performance in the explicit group. All other differences were nonsignificant (all p_Cluster_ > .78).

## Discussion

Learning of regularities—including regularities based on temporal probabilities (most likely time point of target occurrence)—and explicit knowledge about temporal regularities (e.g., due to temporal cueing) are often assumed to maximise performance by actively preparing for certain moments in time at which targets are presented (Nobre & Rohenkohl, 2014). However, previous studies typically utilized unisensory-specific contexts. In this study, we tested whether *explicit knowledge* of temporal regularities would affect temporal expectancies (TE) in different unisensory and multisensory contexts. Furthermore, we tested whether dynamic learning models can be successfully applied to analyse learning of temporal regularities and the temporal dynamics of TE effects.

We found that TEs—in the present study—were most likely to be altered by the modality-related context using a direct model comparison approach. Additional factors such as the dynamics of learning (change of TE effect across runs) and explicit knowledge appear to contribute only minimally to the size of TE effects when considering accuracy and response times. In accord, these findings were further corroborated by parameters derived from learning models. In particular, the probability of answering correctly was relatively stable after an initial spike-like increase in probability and clearly different across modalities, but this difference was stable across all trials (i.e., no modality-specific differences due to learning occurred over time). Further, no consistent overall effect was found for the differences between knowledge groups. Explicit knowledge only improved performance for auditory condition under high modality-specific uncertainty but was also linked to slower learning (shallower learning curves) under low modality-specific uncertainty. Finally, the learning model suggested that performance was modality-dependent; thus, most participants showed no temporal regularity information transfer across conditions.

### Relation to previous results

Our previous results (Ball, Fuehrmann, et al., 2018; Ball, Michels, et al., 2018) suggested that the interaction of TE and modality can be influenced by spatial uncertainty (speakers vs. headphones). After extending the data set, our current results suggest that this additional manipulation by spatial uncertainty contribute only minimally to explaining the data. Nevertheless, the crucial interaction of TE and modality was still supported by the data and noteworthy, when only including accuracies of the best unisensory versus multisensory conditions in the analysis (testing for multisensory interplay), this finding still held true (best model: TE × Modality, evidence ratio other models <.0001). There are several possibilities to explain the enhanced performance in multisensory as compared with unisensory target trials. For example, presenting two stimuli at the same time allows for target identification in one or both modalities. Thus, no matter which modality participants dynamically attend to, target perception would always be possible. While such scenario could explain the multisensory versus unisensory differences in the high modality-specific uncertainty designs (targets embedded in multisensory sequences; thus, unisensory targets might be missed if the other modality is attended), it would not explain the same differences found under low modality-specific uncertainty. Here, unisensory targets were presented in pure unisensory sequences, and thus were always attended. Hence, dynamic shifts of attention in the multisensory sequence should never increase performance as compared with the unisensory conditions; potentially, attentional shifts might rather decrease multisensory performance. Another possibility is that the performance advantage linked to the multisensory condition comes about by integrating the redundant target information (i.e., combining information into a single event). Note, however, that stimuli were not necessarily fully integrated as performance in the audio-visual condition did not differ between the different levels of spatial uncertainty (accuracies: all *F*s < .225, *p*s > .564; RT: all *F*s < 1.63, *p*s > .198); hence, it did not differ between the headphones (sound localised with the head) versus speaker contexts (sound close to visual stimulus). Thus, it is unlikely that auditory and visual stimuli were always bound together in space (i.e., they were perceived from the same locations). Nonetheless, the results indicated that visual and auditory modalities at least interacted in some form with each other independent of the stimulation context (see Spence, 2013, for discussion of the necessity of spatial-alignment in multisensory interactions). These interactions might be based on bidirectional influences of activity in primary sensory areas or early low-level interactions in deeper brain structures (see discussion on neural mechanisms below). Most importantly, the results indicate that the modality context can individually shape whether and how strong TEs are formed.

### Relation of TE and dynamics of learning

State-based learning models have previously been used to successfully determine dynamic learning in contexts such as memory-association tasks, visuo-motor associative learning tasks, and location-scene association (Clarke et al., 2018; Hargreaves et al., 2012; Smith et al., 2004; Wirth et al., 2003). In this study, we evaluated whether they can successfully be applied to temporal pattern learning tasks (i.e., the learning of certain temporal regularities). Our results indicate that the shape of and differences between the modelled learning curves closely resemble effects based on measured average performance. Further, we used the model to formally test our assumption that information about the most likely temporal position is not transferred between modalities (Ball, Michels, et al., 2018). The model supported this claim, even on the individual level (86.5% of all participants) and further suggested that explicit knowledge does not ease the transfer of temporal position knowledge (i.e., the most likely temporal position) across modalities. Importantly, the learning curves also indicate that participants learned the temporal pattern faster in the multisensory compared with unisensory contexts thereby extending the findings of previous reports showing increased performance and TE effects in the multisensory condition (Ball, Fuehrmann, et al., 2018; Ball, Michels, et al., 2018). Potentially, the higher informational content of multisensory stimulation—which has been linked to multisensory performance benefits (e.g., Alais & Burr, 2004; Ball, Fuehrmann, et al., 2018; Ball, Michels, et al., 2018; Driver & Noesselt, 2008; Noesselt et al., 2007; Noesselt et al., 2010; Parise et al., 2012; Starke et al., 2020; Werner & Noppeney, 2010)—also increases speed of extraction of temporal regularities and temporal perceptual learning. Note that the distinction of ‘information transfer vs. no transfer’ between modalities cannot be derived from average performance scores (see Introduction), as these do not capture intertrial relations and dependencies and might be misleading. However, in our study, average scores and results from the learning model still go hand in hand. Together, the results suggest that learning models can provide additional insights into temporal learning, unravel potentially different learning types (if present; here, modality-specific learning), and might also be used to identify the neural origins of temporal regularity learning on a trial-by-trial basis in future neuroimaging studies, as has previously been done in other nontemporal research fields (Clarke et al., 2018; Hargreaves et al., 2012; Smith et al., 2004; Wirth et al., 2003).

### Relation of TE and explicit knowledge

Turning to the differences between explicit and implicit temporal knowledge, previous research suggests that TEs are created implicitly from the temporal trial structure (e.g., rhythms) and from the overall statistical distribution (e.g., increased likelihood for events with a particular foreperiod in one run) of likely events in time by means of statistical learning (Ball et al., 2020; Ball, Fuehrmann, et al., 2018; Ball, Michels, et al., 2018; Breska & Deouell, 2014; Cravo et al., 2013; Rohenkohl et al., 2011; Shen & Alain, 2012). Our results support this assumption as TE effects in our study were largely driven by implicit knowledge (142 out of 200 participants). Thus, statistics about predictable events—at least in our study—seem to be automatically extracted in the majority of participants and to be utilized independently of explicit knowledge about the temporal manipulation.

To our best knowledge, not many studies have directly compared the effects of explicit versus implicit knowledge within the same paradigm. However, a difference in behaviour or neural activation patterns—when based on different experimental paradigms or studies without assessment of participants’ explicit knowledge—would be insufficient to conclude that explicit knowledge truly affects, for example, performance and learning patterns (Ball et al., 2020). And most within-study comparisons of explicitly versus implicitly driven behaviour report the absence of modulating effects due to explicit knowledge. For instance, Max et al. (2015) investigated the impact of distracting sounds (deviant pitch) within a sequence of standard sounds on auditory duration judgments. The authors found no evidence that instructions (explicit vs. no information) altered behavioural performance related to the distraction effect, though they observed changes in the EEG signals (i.e., a lower P3a amplitude when distractors were expected due to instructions). They suggest that differences in neural activity index participants’ involuntary shift of attention to distracting sounds which is altered by prior knowledge (implicit vs. explicit instruction), yet these neural activity changes do not necessarily change the behavioural outcome. In addition, the absence of influences of explicit knowledge on performance was also reported in priming studies (Francken et al., 2011; Van den Bussche et al., 2013), contextual cueing studies (Chun & Jiang, 2003; Geyer et al., 2012; Preston & Gabrieli, 2008; Westerberg et al., 2011) and motor sequence learning tasks (Sanchez & Reber, 2013). Remarkably, results from some studies even indicate that explicit compared with implicit knowledge (1) can be detrimental (Green & Flowers, 2003; Preston & Gabrieli, 2008; Van den Bussche et al., 2013); (2) reduces differences between, for example, cued and uncued trials (Schlagbauer et al., 2012); (3) might only be beneficial on a long-term but not short-term basis (Ewolds et al., 2017) or under very specific task constraints (Stefaniak et al., 2008); and (4) could in principle reflect changes of response strategy rather than a perceptual facilitation (Schlagbauer et al., 2012; Summerfield & Egner, 2009). Recently, we found support for the latter idea of response strategy shifts in a simple visual TE paradigm (Ball et al., 2020). We term this paradigm ‘simple’, as accuracies were close to ceiling and the cue was only followed by the target, rendering it easily identifiable as the stimulus that ‘has to be responded to’. Here, we extent our previous findings to complex multisensory TEs paradigms (targets hidden among distractors) by showing that explicit knowledge has only a marginal effect on performance, which is also not always favourable.

In general, we only found weak evidence for differences between participants with explicit and implicit knowledge. In the experiments with high modality-specific uncertainty, accuracies were enhanced in the explicit group, but only in the auditory condition as determined by the learning curve analysis. Note that our previous reports showed that the unimodal auditory condition, was the preferred condition (i.e., unimodal condition with best performance across all unimodal conditions) for most of participants (Ball, Fuehrmann, et al., 2018). Thus, explicit knowledge might potentially help to overcome response conflicts by identifying and discriminating the unisensory target in a multisensory stimulus sequence, but only for the preferred unisensory modality. In accord, 19% of the explicit participants made their statement specifically about the auditory condition lending further support to the notion that learning occurred modality-specific. Given our results, it is possible that explicit knowledge is ineffective whenever there is no conflict (audio-visual target) or the unisensory temporal position is less known or even unknown (visual target). Another possibility is that explicit knowledge enhances processing of the stimulus, but only in sensory systems with high temporal acuity (i.e., the auditory system; see e.g., Ball, Michels, et al., 2018; Nobre & Rohenkohl, 2014, for discussion of thie issue). However, if this would be true, we would expect to find similar results for the auditory targets under low modality-specific uncertainty, which was not the case. In contrast, the learning curves for the low modality-specific uncertainty experiments even showed a trend for lower performance in the explicit knowledge group. Following up on this pattern of results, it might be that explicit knowledge does mainly help to *detect* the target more reliably in time—thereby increasing the confidence about target presence at certain points in time—while explicit knowledge does (on average) not help to finally *discriminate* the target more reliably. However, such increase in confidence might not always be beneficial and result in premature responses, thereby sacrificing accuracy in discrimination tasks, especially under easy task regimes; an interpretation which would be in line with our previous report (Ball et al., 2020) and other findings (Schlagbauer et al., 2012; Summerfield & Egner, 2009). Under more difficult task regimes, explicit knowledge might help to better cope with the task, at least for specific unisensory conditions and independently of the run-wise manipulation of temporal regularities.

Finally, it is worth discussing three potential reasons why explicit knowledge was ineffective to sufficiently alter performance. First, explicit knowledge (without prior instructions by the experimenter) is a continuum. Thus, while some participants might exhibit ‘strong’ explicit knowledge (“The target appears after 400 ms”), others might exhibit only ‘vague’ explicit knowledge (“The target occurs more likely early in the sequence”). The overall strength of explicit knowledge within the group might determine whether performance differs from the implicit group. However, 41 out of the 58 participants with explicit knowledge, had strong explicit knowledge rendering it less likely that the absence of differences between the explicit and implicit groups is driven by the strength of explicit knowledge. Still, direct manipulations of explicit knowledge (by informing participants about temporal regularities) might maximise differences between implicit and explicit knowledge groups (although even this manipulation might be ineffective; see Ball et al., 2020). Second, as participants build and update their explicit knowledge throughout the experiment, effects of explicit knowledge might be stronger at the end than the beginning. However, our analyses (which took this factor into account) did not provide evidence for this suggestion either. Finally, it might be that learning in the implicit and explicit group partially or even fully overlaps in the sense that learning of temporal regularities is always based on automatic, incidental learning and automatic deployment of attention. Hence, explicit knowledge might only imply that participants became aware of the learned entity (here, temporal regularities) without being able to use this information to actively shift temporal attention (see also our discussion on potential underlying neural mechanisms below).

Optimally, future tasks would incorporate a measure of detection as well as discrimination performance (without priming the temporal task component) and potentially also confidence ratings to further elucidate the interplay of confidence, detection, discrimination, and explicit knowledge in TE tasks. Additionally, the differences between groups appear to be context sensitive—they depend on the modality context as well as task difficulty or rather task design. Thus, future studies should also systematically study the influence of these factors to allow forming theories about how TE, modality, distraction and explicit knowledge holistically affect performance.

### Relation of TE, dynamics of learning, and explicit knowledge

We hypothesized that temporal regularity learning might elevate performance with repeated exposure to temporal regularities (change of the size of TE across runs); this assumption was based on results from contextual cueing, perceptual, and motor sequence learning studies (Bueti & Buonomano, 2014; Chun & Jiang, 1998; Clegg et al., 1998). They reported improved performance for repeated (i.e., expected) compared with new (i.e., unexpected) layouts and sequences. However, TE effects were not sufficiently modulated by repeated exposure, neither for response times nor accuracies, as indexed by the model comparison. This might be due to an information transfer across runs. Hence, when participants learned to attend the early position in one run, they slowly shifted their attention away from the early position in the next run, resulting in similar TE effect sizes across runs. Moreover, learning curves only showed minimal accuracy fluctuations over time (see learning curve results). Most importantly, the largest effect we found—the performance difference between modalities—was present in each trial; hence, this difference between modalities did not require to learn, for example, a certain moment in time to facilitate performance. We further hypothesised that explicit knowledge might be based on or related to faster learning; however, as mentioned above, learning speed was similar in the explicit and implicit group. Thus, it appears that effects of explicit knowledge on learning and vice versa as well as TE effects (based on shifts of attention due to run-wise probability manipulations) are not affected by run-wise dynamics of learning over the course of an experiment. At this point it remains open whether learning effects on TEs are, for example, more pronounced in long-term studies (experiments over several weeks). Nevertheless, based on our data, we conclude that short-term exposure to temporal regularities within one experiment (here, less than 3 hours) has little effect on the size of TEs in complex task designs.

### Possible limitations

A possible limitation of the current study is the assessment of implicit and explicit knowledge of temporal regularities. In other research fields like contextual cueing (Chun & Jiang, 1998), explicit knowledge is typically tested by comparing recognition accuracy of old versus new scenes. However, it is currently debated whether these post hoc detection tests truly supports the null hypothesis (i.e., implicit knowledge) or whether the assessments of implicit versus explicit knowledge by means of recognition tests in previous studies were underpowered (Vadillo et al., 2016). Furthermore, it is difficult to design a perceptual post hoc test for recognising certain temporal delays. While deciding whether a certain stimulus (e.g., a car or house) has been presented before (is old or new) results in either correct or incorrect responses; deciding whether a duration has been presented before results in ‘more or less correct’ responses. For instance, our targets were always presented after 400 ms and 1,600 ms. Presenting a 450 ms stimulus (a dot, square, sound, etc.) and asking participants whether the presented duration matched the time interval after which targets were presented might result in a ‘yes’ response. While it is incorrect that targets were presented after 450 ms, such judgement would also not be far of the truth. Thus, it is unclear how to handle such responses. Furthermore, there might be trial-dependencies additionally influencing participants duration judgement (Wehrman et al., 2020), the stimulus material used for the post hoc test might influence how precise duration judgments are (Ball, Michels, et al., 2018) and even for the auditory modality, duration judgements are never perfect (namely, above-chance performance requires duration difference of tens of milliseconds and ceiling performance requires differences of hundreds of milliseconds; see Morrongiello & Trehub, 1987). Hence, all these factors would affect participant’s judgement, rendering the responses an unreliable test of explicit knowledge. More importantly, every condition can only be presented once in postexperimental awareness tests to avoid response biases (presenting old stimuli repeatedly could result in priming participants to respond to these stimuli as “old”), leaving two trials (early and late temporal position) in the “old” condition for statistical analyses which is insufficient. The same problems apply when using our employed paradigm and test for old versus new temporal target positions as, for example, individual temporal positions (e.g., third vs. fifth position in stimulus stream) are hardly distinguishable due to the fast presentation frequency (Ball, Michels, et al., 2018); and again, the trial number for testing recognition of the old temporal positions would be insufficient.

Instead, we opted for using an exhaustive post hoc questionnaire (Ball et al., 2020; Ewolds et al., 2017; Heuer & Schmidtke, 1996; Nissen & Bullemer, 1987) and motivated participants to even guess the most likely target positions. Nonetheless, this procedure still does not allow to pinpoint the exact time when participants gathered explicit knowledge. To quantify the onset of learning, we chose a computational model which was specifically designed to have higher sensitivity in capturing the ‘true’ learning onset and to test for difference across conditions (Smith et al., 2004); further, the model was successfully applied in previous nontemporal studies (Clarke et al., 2018; Hargreaves et al., 2012; Smith et al., 2004; Wirth et al., 2003). However, assuming that the learning trial represents the speed of learning, we did not find evidence for earlier learning onsets in the explicit group. Thus, if the learning onset in the explicit group represents participants becoming aware of the temporal position, this did not happen faster than implicit learning of temporal position in the implicit group. Hence, this finding might imply that learning onsets in both groups were rather based on implicit statistical learning and that participants noticed the temporal regularity later on in the experiment.

Another limitation might be based on our choice of experimental designs. As the time point of target occurrence is always based on a certain position within the stimulus sequence, participants might not create interval scaled TEs (estimates of time) but rather ordinally scaled expectations (estimates of position, e.g., by counting). It is under debate, whether estimates due to counting and time are principally mediated by different, overlapping, or even the same neural mechanism (Balci & Gallistel, 2006; Fetterman & Killeen, 2010; Meck et al., 1985; Meck & Church, 1983; Whalen et al., 1999). Noteworthy, most theories about timing assume that e.g., an internal clock (be it modality specific or amodal) accumulates beats (e.g., firing of neurons) to estimate durations (Gibbon, 1991; Gibbon et al., 1984; Treisman, 1963), or that our environment, including temporal regularities, are sampled periodically or discretely (for recent review, see Nobre & van Ede, 2017). Thus, one could argue that processing of temporal information is always based on counting, irrespective of the type of stimulation (for more indepth discussion of counting vs. timing, see Ball, Michels, et al., 2018). However, we recently showed that the effects presented here hold true and were virtually identical under higher stimulation frequencies (10 Hz and 15 Hz instead of 5 Hz stimulus streams; see Ball, Nentwich, & Noesselt, 2021). As these higher stimulation frequencies render stimuli virtually uncountable, it is rather likely that even in the present design, participants form expectation about points in time and not positions.

### Possible relevance for time-based expectations

In this last part, on helpful anonymous reviewers’ requests, we would like to discuss how our results relate to two important topics: (1) time-based event expectations and (2) neural mechanisms of TEs.

Firstly, our results might indicate that participants created ‘time-based event expectations’ (TBEE) instead of TEs. TEs are solely based on temporal features (which time point is more likely) and in principle should facilitate performance irrespective of target’s identity. However, TBEEs are expectations for certain features/events conditioned upon specific points in time. For example, after hearing a knock on the door, it is likely that someone enters the room, but the longer the door stays closed, we start to expect that they did not hear us inviting them in. TBEEs have mainly been investigated with simple visual shapes, coloured numbers, and affective information (Aufschnaiter, Kiesel, Dreisbach, et al., 2018; Aufschnaiter, Kiesel, & Thomaschke, 2018; Kunchulia et al., 2017; Thomaschke et al., 2018; Thomaschke & Dreisbach, 2015; Thomaschke et al., 2011; Volberg & Thomaschke, 2017), by, for example, presenting a circle with 80% probability after a short cue–target delay (e.g., 600 ms), while a presentation of a square is 80% probable after a long cue–target delay (e.g., 1400 ms). Note that in these experiments each time point of target occurrence typically has the same likelihood (while they are unevenly distributed in TE studies). Like in TE studies, this line of research shows that, mainly, response times improve—but here for likely compared with unlikely time-event couplings—and that these time-event couplings can be learned implicitly.

Given our results, the question arises, whether TE and TBEE are actually different processes. Here, we employed a ‘true’ TE design, in the sense that—given our design—event types (AV, A, V) were distributed equally across time-points, while the time points of target occurrence themselves were unevenly distributed (e.g., more early than late trials). Thus, there was no time-event contingency in our paradigm. Still, we observed differences in TE effect sizes across modalities and our learning models suggest that information is not transferred across modalities and that learning was faster in the AV condition. Hence, our results might imply that participants created TBEEs—they started to expect targets not only to occur at a certain time point but this expectation was also context driven; expectations were stronger for some modalities and weaker for other depending on the paradigm. However, if the strength of expecting a certain modality at a certain point in time implies the presence of TBEEs, then unisensory TE paradigms are a special case of TBEEs (100% modality expectation). To investigate behavioural patterns of true multisensory TBEE (i.e., each foreperiod was equally likely), we recently conducted a study in which we only manipulated target-foreperiod contingencies (hence, TBEE) of auditory and visual targets (Ball, Andreca, & Noesselt, 2021). TBEEs were quantified as the performance difference between the primary and secondary targets (e.g., short foreperiod 80% auditory [primary] and 20% visual targets [secondary]) at each foreperiod. Importantly, we found that even audio-visual TBEEs—similarly to multisensory TEs—are context-specific. Not only were the effects (higher performance for primary modality after short or long foreperiod) stronger for the auditory compared with the visual modality, the direction of the effect after each foreperiod was mainly driven by expectations for the early auditory target. Whenever the expectations matched with the context (auditory early, visual late), primary target performance was facilitated. Whenever there was a mismatch with the context (visual early, auditory late), participants created expectations in the opposite direction (towards the secondary target after each foreperiod), which strengthened over time. As we argued in our recent paper, depending on whether stimuli compete for interest, the one better suited for the task (e.g., auditory stimuli in temporal tasks and visual stimuli in spatial tasks) potentially wins the race. Given the similarity of results across our studies (for further discussion, see Ball, Andreca, & Noesselt, 2021), TE and TBEE might be a single process (namely, always TBEE), in which the context simply determines for which target class temporal regularities are learned and how strong TEs are formed (e.g., unisensory visual paradigm: TE effect for visual; mixed paradigm with auditory and visual: TE(A) > TE(V)).

### Possible relevance for underlying neural mechanisms

Another way to interpret our results only in the context of TEs is related to the neural implementation of TE. Let us first start with the assumption of one single internal pacemaker (Gibbon, 1991; Gibbon et al., 1984; Treisman, 1963), a central clock, a dedicated time network that orchestrates and directs temporal attention and TEs. One could put forward a bottom-up/top-down mechanism in which perception of targets at certain points in time (bottom-up) establishes TEs (due to statistical learning) in a parietal control structure or the basal ganglia (for review, see Coull et al., 2011), which in turn modulates (top-down; e.g., low-level) unisensory (Jaramillo & Zador, 2011; Kikuchi et al., 2017; Lima et al., 2011; Turk-Browne et al., 2010) and motor areas. Such interplay of hierarchically higher and lower level areas might result, for instance, in different states of entrainment in primary visual and auditory cortices (Cravo et al., 2013; Lakatos et al., 2008; Lakatos et al., 2009; C. E. Schroeder et al., 2008) and in motor areas which potentially improve target discrimination and/or might affect response preparation (Coull & Nobre, 1998).

However, it is possible that modality-specific timing networks rather than a central control structure mediate the effects presented in our study. Accordingly, implicit learning as well as TEs have been linked to activity in primary auditory and visual cortex (Jaramillo & Zador, 2011; Kikuchi et al., 2017; Lima et al., 2011; Turk-Browne et al., 2010) in different mammals. Hence, it is possible that low-level sensory-specific areas serve as modality-specific temporal networks without central control. As mentioned before, the auditory domain has a higher temporal precision and dominates in temporal tasks (Burr et al., 2009; Fendrich & Corballis, 2001; Hromádka & Zador, 2009; Recanzone, 2003; Repp & Penel, 2002; Welch et al., 1986), which would be in accord with the finding that TE effects were less pronounced in the visual condition (high modality-specific uncertainty).

Differences between unisensory and multisensory target conditions can be explained in two ways without relying on a central clock. One could assume that precision for target discrimination is improved by direct connections between primary cortices (Driver & Noesselt, 2008). Whenever auditory and visual cortex receive input, entrainment in one area might directly interact with entrainment in the other area, thereby increasing the reliability/robustness of processed stimulus information and facilitating the extraction of temporal regularities. Another possibility is that multisensory performance improvements are mediated by areas implicated in the processing of multisensory but also temporal information which would ease potential interaction of these factors. Possible candidate areas for multisensory interplay would be, for example, the posterior parietal cortex (Coull et al., 2011; Rohe & Noppeney, 2016) as well as the posterior superior temporal sulcus (Driver & Noesselt, 2008; Marchant et al., 2012; Noesselt et al., 2007; Noesselt et al., 2010); areas which have been linked to enhanced processing of synchronous audio-visual stimuli and have also been implicated in perceptual and implicit learning (Powers et al., 2012; Tzvi et al., 2016). Given that all aforementioned areas are implicated in implicit learning, it is possible that the processing of temporal information in these areas is not only modality-specific but also automatic. This would be in line with a large part of the literature indicating that TEs are created automatically/implicitly (for review, see Nobre & Rohenkohl, 2014), including our own results presented here (see also Ball et al., 2020; Ball, Fuehrmann, et al., 2018; Ball, Michels, et al., 2018). However, please note that here we did not collect neuroimaging data, and additional experiments are required to test the abovementioned alternatives.

To close, if temporal information is mainly processed automatically, this could explain why differences between participants with explicit and implicit perception are largely absent in this and in our previous study (Ball et al., 2020). In such scenario, the processing would be the same for both groups, resulting in similar performance, while only their awareness of the manipulation is different. This would also imply that low-level processing mechanisms and stimulus perception might be impenetrable by explicit knowledge and cognition (Firestone & Scholl, 2015). In this sense, explicit knowledge could not be used to actively shift attention to 400 ms after stimulus onset. Rather, explicit knowledge might be used to utilize individual strategies: under high modality-specific uncertainty one could, for example, attend more to one than the other stimulus stream, which would explain the increased auditory performance for participants with explicit knowledge. Under low modality-specific uncertainty, participants with explicit knowledge might not be willing to wait till the end of the sequence to confirm their initial percept of the target stimulus (as the target stimulus was likely already presented), thus potentially sacrificing performance which would fit the observed pattern of results. Additionally, our previous report suggested that providing explicit information about the temporal manipulation resulted in a speed–accuracy trade-off rather than perceptual facilitation (Ball et al., 2020), which would be in line with the ineffectiveness of explicit knowledge to manipulate low level neural processing in our experiments.

## Conclusion

Here we show—using a computational learning model—that temporal regularities were learned separately for each modality and that explicit knowledge did not ease the transfer of information across modalities. Thus, knowing when a target is likely to occur is not automatically generalised. Further, it is often suggested that explicit knowledge changes the recruitment of cognitive resources. However, our results suggest that top-down modulations of behaviour due to explicit knowledge appear to be detrimental under low task difficulty (in line with previous reports) and only facilitated unisensory auditory target performance when the target modality in a given trial was unpredictable (high task difficulty). Thus, explicit knowledge might partially help to resolve response conflicts under high uncertainty but might render participants overconfident (while sacrificing accuracy) when targets are more easily detectable. Additionally, audio-visual stimulation resulted initially in faster and better learning. However, the size of TE effects did not significantly change across runs, most likely due to reversal learning at the beginning of each run. Hence, dynamics of temporal learning throughout the whole experiment only contributed little to the observed TE effects (i.e., no continuous increase in TE effect size across runs). Together, the results suggest that learning of temporal regularity might be more reliable in multisensory than unisensory context and that explicit knowledge as well as dynamics of learning have little to no beneficial effect on TE. Given our sample size and the comparatively small effects, our results also stress the importance to carefully interpret changes in behaviour due to explicit knowledge when assessed by across-study designs.

## Supplementary Information

ESM 1 (PDF 833 kb)

ESM 2 (XLSX 554 kb)

## Footnotes

1 Note that we, like others, operationalise implicit knowledge as being unaware of the learned experimental manipulation (here, manipulations of temporal regularities) while participants with explicit knowledge either become aware of the manipulation during the experiment or were informed about the manipulation beforehand (Ball et al., 2020; Batterink et al., 2015; Droit-Volet & Coull, 2016; Ellis, 2009; Frensch & Rünger, 2003; Reber, 1976; Turk-Browne et al., 2010).

2 To illustrate the potential limits of conclusions derived from average scores versus trial dynamics, imagine a trial sequence in which Conditions A and B are always presented alternatingly (A-B-A-B-…). Now, imagine that a correct/incorrect response for B is 100% dependent on the preceding response for A (Scenario 1). In contrast, now imagine we simply shuffle the scores of B across B’s trials (increasing trial-by-trial independence; Scenario 2). In both scenarios the sequence (A-B-A-B-…) as well as the average performance of conditions are identical. Importantly, however, the underlying learning mechanism is either dependent (information transfer; Scenario 1) or independent (no information transfer; Scenario 2). Now, imagine, for example, that in only 80% of successive trials, responses are identical in A and B (new Scenario 1); hence, their average performance scores potentially differ. Again, imagine that we shuffle the scores of Condition B (new Scenario 2). In both scenarios, we have again the same condition sequence and average performance scores—now different between A and B—but the underlying learning mechanisms are again either dependent (information transfer; Scenario 1) or independent (no information transfer across trials; Scenario 2). These examples clearly illustrate why additional analyses and models are required to make conclusions about, for example, whether learning is generalised across sensory systems, while average scores are insufficient to draw conclusions about underlying learning mechanisms.

3 Note that information transfer between runs might be driven by the experimental design. If temporal regularities do not change across runs, information might always be transferred from one run to the next. However, changing probabilities of target’s temporal positions across runs (Ball, Fuehrmann, et al., 2018; Ball, Michels, et al., 2018; Jaramillo & Zador, 2011) might either result in information transfer and potential reversal learning (new probable position has to be learned and the old one discounted) or in a complete reset of learning at the beginning of each run due to an unstable environment (i.e., constant change of temporal regularities). Note that information transfer or learning reset could also happen within one run, when, for example, the first run-half incorporates different temporal regularities than the second half.

4 Most TE tasks are rather simple, and findings are mainly based on reaction times (see Nobre & Rohenkohl, 2014). For instance, the target in rhythm tasks is often highlighted (e.g., by colour changes of placeholders) and in cueing tasks, the target is the only stimulus following the cue. Thus, participants always know which stimulus to respond to. This is often not the case for real-life situations, where important information is embedded among irrelevant information. Making the aforementioned tasks more difficult, requires, for example, to change the stimulus contrast. However, this might add more conditions (different contrast values), require a lengthy threshold adjustment (which, if the task is too easy, might not result in stable performance), or it might be impossible to adjust thresholds if they are below the limits of the presentation equipment. This might also be the reason for the prevalent analyses of RTs in the TE literature. One way to shift accuracy below ceiling is to make the paradigm more complex by adding distracting information (S. C. Y. Schroeder et al., 2018). In turn, task difficulty is constantly increased due to a reduction of target’s identifiability, thus rendering perception of its temporal position also less reliable. Additionally, if the experiment is too simple, participants might easily become aware of the temporal manipulation (for discussion, see Ball et al., 2020). Hence, distractors can also be used to ‘mask conscious access’ to temporal regularities, thereby increasing the chance to find participants with implicit knowledge. To this end, we adapted the design introduced by Jaramillo and Zador (2011), originally used to test TE in rats, which met all the abovementioned requirements for analysing RT, accuracy, and potential differences between participants with implicit and explicit knowledge.

5 Note that we used two positions only, as this is the minimum number of positions required to induce a TE effect and likely increases the chance to find TE effects, as variability of possible temporal target positions is reduced to a minimum. Adding more positions would either (1) increase the time required to complete the experiment *n*-fold (*n* = number of added positions; current completion time: 2.5–3 h) or (2) reduce the quality of behavioural estimates when trial numbers are lowered. Further, TE effects at each position would potentially be reduced and statistical power would be lowered due to adding a new factor to the analysis (early target position). Moreover, qualitatively similar yet smaller TE effects have been found in a similar paradigm when more than one target position (e.g., 2, 3, 4) was used (Ball, Michels, et al., 2018).

## Declarations

### Data availability statement

The authors declare that the analysed data as well as potential supplementary analyses (not reported in the main manuscript) are available in the supplementary information files.

### Code availability

Data were analysed with R/Rstudio (freely available) and the Learning Curve Analysis software package for MATLAB (http://www.neurostat.mit.edu/behaviorallearning). The remaining code is available upon request.

### Open Practices Statement

The data and materials are available in the supplementary information files and upon request (e.g., codes). None of the experiments was preregistered.
