# Supplementary material for: Minimal interplay between explicit knowledge, dynamics of learning and temporal expectations in different, complex uni- and multisensory contexts
Source: Atten Percept Psychophys. 2021 May 11;83(6):2551–73. doi: 10.3758/s13414-021-02313-1 (PMC8302534; doi:10.3758/s13414-021-02313-1)
Supplement: Supplementary file 1 — (PDF 833 kb) [file 13414_2021_2313_MOESM1_ESM.pdf]

Supplement: Abbreviations and main concepts 2
Supplement 1: Re-analysis Ball et al. (2018) 3
A1: Experiment 1 - rm ANOVA (accuracy scores) 3
A3: Experiment 3 - rm ANOVA (accuracy scores) 5
A4: Experiment 4 - rm ANOVA (accuracy scores) 6
B1: Experiment 1 - rm ANOVA (RT scores) 7
B2: Experiment 2 - rm ANOVA (RT scores) 8
B3: Experiment 3 - rm ANOVA (RT scores) 9
B4: Experiment 4 - rm ANOVA (RT scores) 10
Supplement 2: Mixed Model (MM) design in R 11
A1: MM for accuracy & RT data (InputData = whole data set) 11
A2: MM for accuracy & RT data (InputData = low OR high modality-spec. uncertainty) 11
B1: MM for learn trial data (InputData = whole data set) 12
B2: MM for learn trial data (InputData = low OR high modality-spec. uncertainty) 12

Supplement: Abbreviations and main concepts

Abbreviations

A – auditory
Acc – accuracy
AIC – Akaike Information criterion
AV – audio-visual
Know – knowledge group
RT – response times
Sp. Unc – spatial uncertainty
TE – temporal expectations
TBEE – time-based event expectations
V – visual

Explicit/implicit knowledge

Participants with implicit knowledge process an experimental manipulation without being aware of it. For instance, they might implicitly learn that a target occurs more often after a short than long cue-target delay or more often on the left than right side of the display. Participants with explicit knowledge either became aware of the manipulation over the time course of the experiment or they were informed about the manipulation (e.g. during the instruction).

Explicit/implicit timing tasks

This is not to be confused with explicit/implicit knowledge. In explicit timing tasks, participants have to actively judge time in some form (e.g. by deciding which stimulus was presented longer). In implicit timing task, time is manipulated orthogonally to a primary task (e.g. judging the colour or frequency of a stimulus). Thus, time has no direct implication for the task at hand. However, it is also often assumed that temporal information in implicit timing tasks is processed implicitly (implicit knowledge). As this assumption is rarely tested and participants might become aware of the manipulation during the experiment, results of implicit timing tasks might be confounded.

Multisensory interplay/interaction

The presentation of two or more stimulus modalities (typically synchronously) results in improved performance compared to the best unisensory modality (highest accuracy or lowest reaction time).

Multisensory Integration

Similar behavioural effects as for multisensory interaction. But here the different modalities are integrated into a single percept.

Temporal expectations

Expectations for one or more specific points in time, which are more likely than all other time points. Temporal expectations should facilitate performance irrespective of targets identity at the expected time point.

Time-based expectations

Expectations for a certain stimulus (e.g. a square) that are conditioned on a certain point in time (e.g. a square is more likely appearing at 400 than 2000 ms after cue onset). Time-based expectations should facilitate performance for the most likely event-time contingency.

Supplement 1: Re-analysis Ball et al. (2018)

*Description: Given that we do not exclude, but transform outliers in the current study (conversion of trials with < 150 ms RT to incorrect trials), we re-analysed all experimental data using this criterion to check whether it would change any results. As in Ball et al. (2018) mean accuracies were converted to d-prime and ANOVAs were conducted for each experiment and performance measure (A1-4: d-prime, B1-4: RT). The results are virtually identical with our previous report. Note that we also show plots of the TE*Modality data (with 95% confidence intervals as calculated in JASP). Abbreviations: df = degrees of freedom, F = F-statistic, t = t-statistic, p/pbonf = p value/p value Bonferroni corrected, η² = eta-squared, TE = temporal expectation, AV = audio-visual, A = auditory, V = visual, C1/C2 = contrasted conditions in post hoc test.*

A1: Experiment 1 - rm ANOVA (accuracy scores)

effect	Sphericity Correction	df	F	p	η²
TE	-----	1	28.291	< .001	0.043
Residual	-----	29			
Modality	GG	1.297	2.495	0.115	0.065
Residual	GG	37.627			
TE ∗ Modality	GG	1.659	0.655	0.496	0.002
Residual	GG	48.118			

Post hoc comparison for main effect TE and main effect Modality (post hoc effects in JASP)

C1	C2	Mean Difference	SE	t	p_{bonf}
Unexpected	Expected	-0.167	0.031	-5.319	< .001
AV	A	0.246	0.113	2.183	0.099
	V	0.169	0.113	1.501	0.417
A	V	-0.077	0.113	-0.683	1

Data plot TE * Modality (individual curves are expected/unexpected trials)

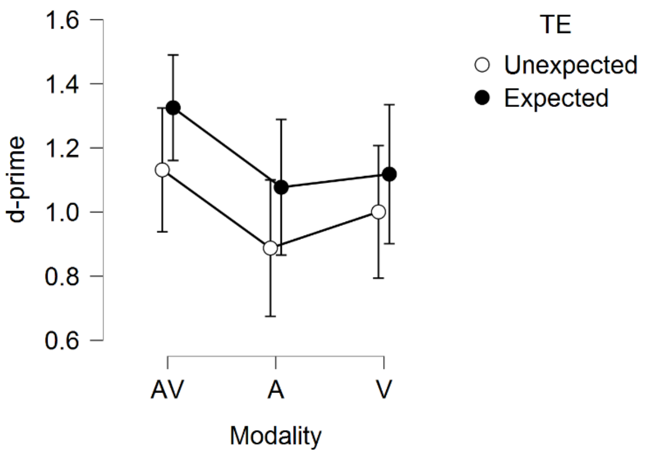

A2: Experiment 2 - rm ANOVA (accuracy scores))

effect	Sphericity Correction	df	F	p	η²
TE	-----	1	27.286	< .001	0.078
Residual	-----	29			
Modality	GG	1.467	9.14	0.001	0.166
Residual	GG	42.55			
TE ∗ Modality	-----	2	6.705	0.002	0.027
Residual	-----	58			

Post hoc comparison for main effect TE and main effect Modality (post hoc effects in JASP)

C1	C2	Mean Difference	SE	t	p_{bonf}
Unexpected	Expected	-0.245	0.047	-5.224	< .001
AV	A	0.308	0.102	3.022	0.011
	V	0.421	0.102	4.13	< .001
A	V	0.113	0.102	1.109	0.816

Post hoc comparison for factor TE (expected vs. unexpected) for each modality type (simple main effects in JASP)

Modality	df	F	p
AV	1	24.14	< .001
A	1	14.593	< .001
V	1	0.63	0.434

*Data plot TE * Modality (individual curves are expected/unexpected trials)*

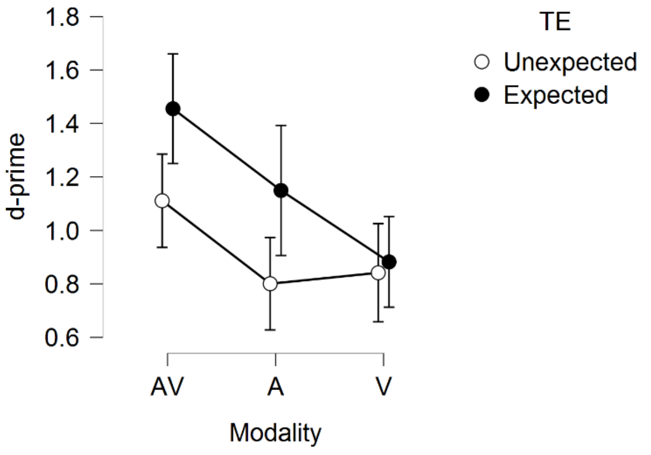

A3: Experiment 3 - rm ANOVA (accuracy scores)

effect	Sphericity Correction	df	F	p	η^2
TE	-----	1	19.721	< .001	0.027
Residual	-----	29			
Modality	GG	1.339	17.564	< .001	0.317
Residual	GG	38.839			
TE ⁎ Modality	-----	2	0.157	0.855	5.087e -4
Residual	-----	58			

Post hoc comparison for main effect TE and main effect Modality (post hoc effects in JASP)

C1	C2	Mean Difference	SE	t	p_{bonf}
Unexpected	Expected	-0.143	0.032	-4.441	< .001
AV	A	0.522	0.102	5.144	< .001
	V	0.52	0.102	5.122	< .001
A	V	-0.002	0.102	-0.021	1

*Data plot TE * Modality (individual curves are expected/unexpected trials)*

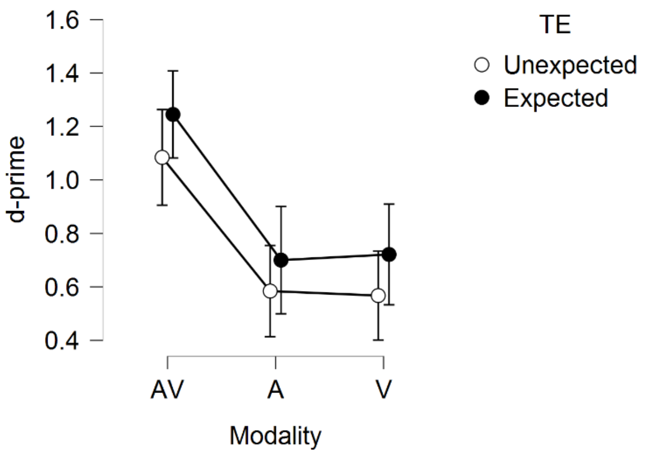

A4: Experiment 4 - rm ANOVA (accuracy scores)

effect	Sphericity Correction	df	F	p	η²
TE	-----	1	7.238	0.012	0.012
Residual	-----	29			
Modality	GG	1.551	18.843	< .001	0.34
Residual	GG	44.985			
TE ⁎ Modality	-----	2	3.628	0.033	0.009
Residual	-----	58			

Post hoc comparison for main effect TE and main effect Modality (post hoc effects in JASP)

C1	C2	Mean Difference	SE	t	p_{bonf}
Unexpected	Expected	-0.093	0.034	-2.69	0.012
AV	A	0.489	0.099	4.935	< .001
	V	0.558	0.099	5.629	< .001
A	V	0.069	0.099	0.694	1

Post hoc comparison for factor TE (expected vs. unexpected) for each modality type (simple main effects in JASP)

Modality	df	F	p
AV	1	12.043	0.002
A	1	2.179	0.151
V	1	0.034	0.856

*Data plot TE * Modality (individual curves are expected/unexpected trials)*

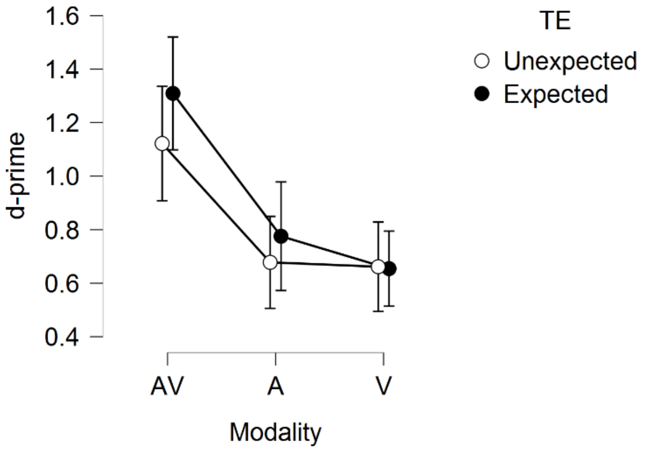

B1: Experiment 1 - rm ANOVA (RT scores)

effect	Sphericity Correction	df	F	p	η²
TE	-----	1	33.453	< .001	0.091
Residual	-----	29			
Modality	GG	1.335	4.011	0.041	0.094
Residual	GG	38.718			
TE ∗ Modality	-----	2	5.13	0.009	0.009
Residual	-----	58			

Post hoc comparison for main effect TE and main effect Modality (post hoc effects in JASP)

C1	C2	Mean Difference	SE	t	p_{bonf}
Unexpected	Expected	0.127	0.022	5.784	< .001
AV	A	-0.143	0.056	-2.546	0.041
	V	-0.131	0.056	-2.347	0.067
A	V	0.011	0.056	0.199	1

Post hoc comparison for factor TE (expected vs. unexpected) for each modality type (simple main effects in JASP)

Modality	df	F	p
AV	1	26.026	< .001
A	1	37.042	< .001
V	1	6.569	0.016

*Data plot TE * Modality (individual curves are expected/unexpected trials)*

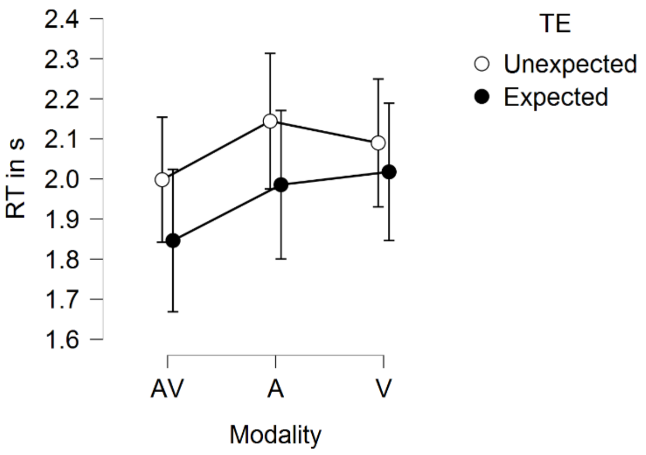

B2: Experiment 2 - rm ANOVA (RT scores)

effect	Sphericity Correction	df	F	p	η^2
TE	-----	1	20.999	< .001	0.163
Residual	-----	29			
Modality	GG	1.254	5.587	0.017	0.087
Residual	GG	36.354			
TE ⁎ Modality	-----	2	5.794	0.005	0.013
Residual	-----	58			

Post hoc comparison for main effect TE and main effect Modality (post hoc effects in JASP)

C1	C2	Mean Difference	SE	t	p_{bonf}
Unexpected	Expected	0.142	0.031	4.582	< .001
AV	A	-0.1	0.038	-2.621	0.034
	V	-0.118	0.038	-3.107	0.009
A	V	-0.018	0.038	-0.486	1

Post hoc comparison for factor TE (expected vs. unexpected) for each modality type (simple main effects in JASP)

Modality	df	F	p
AV	1	16.63	< .001
A	1	20.51	< .001
V	1	12.82	0.001

*Data plot TE * Modality (individual curves are expected/unexpected trials)*

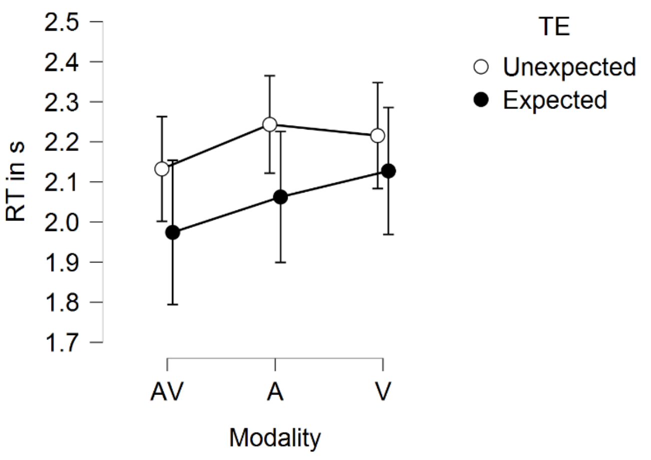

B3: Experiment 3 - rm ANOVA (RT scores)

effect	Sphericity Correction	df	F	p	η²
TE	-----	1	18.272	< .001	0.052
Residual	-----	29			
Modality	GG	1.367	20.232	< .001	0.342
Residual	GG	39.63			
TE ⁎ Modality	-----	2	1.366	0.263	0.001
Residual	-----	58			

Post hoc comparison for main effect TE and main effect Modality (post hoc effects in JASP)

C1	C2	Mean Difference	SE	t	p_{bonf}
Unexpected	Expected	0.098	0.023	4.275	< .001
AV	A	-0.208	0.048	-4.307	< .001
	V	-0.3	0.048	-6.207	< .001
A	V	-0.092	0.048	-1.901	0.187

*Data plot TE * Modality (individual curves are expected/unexpected trials)*

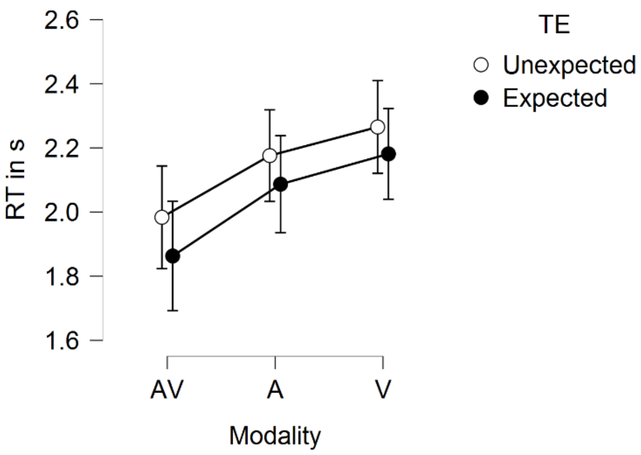

B4: Experiment 4 - rm ANOVA (RT scores)

effect	Sphericity Correction	df	F	p	η^2
TE	-----	1	15.156	< .001	0.047
Residual	-----	29			
Modality	GG	1.507	17.253	< .001	0.304
Residual	GG	43.717			
TE ∗ Modality	-----	2	1.015	0.369	0.002
Residual	-----	58			

Post hoc comparison for main effect TE and main effect Modality (post hoc effects in JASP)

C1	C2	Mean Difference	SE	t	p_{bonf}
Unexpected	Expected	0.082	0.021	3.893	< .001
AV	A	-0.206	0.044	-4.721	< .001
	V	-0.235	0.044	-5.388	< .001
A	V	-0.029	0.044	-0.667	1

*Data plot TE * Modality (individual curves are expected/unexpected trials)*

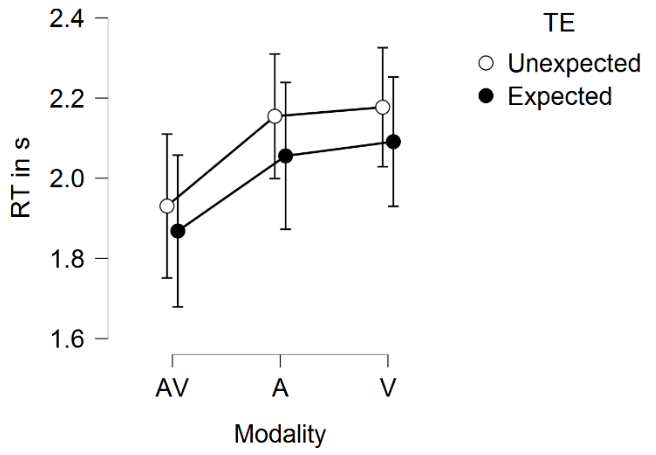

Supplement 2: Mixed Model (MM) design in R

Description: R code for computing models for different performance measures.

A1/2: models for accuracy and RT (split for whole data set and modality-specific analyses)
B1/2: models for learning trials (split for whole data set and modality-specific analyses)

Perf: accuracy, RT oder learn trial scores
TE: Temporal Expectation - factor with 2 levels (1 = no, 2 = yes)
Mod: Modality - factor with 3 levels (1 = AV, 2 = A, 3 = V)
SpatUnc: Spatial Uncertainty - factor with 2 levels (1 = low, 2 = high)
Know: Knowledge group - factor with 2 levels (1 = implicit, 2 = explicit)
Run: Run (which part of experiment) - factor with 3 levels (1 = run1/2, 2 = run3/4, 3 = run5/6)
MSUnc : Modality-specific Uncertainty - factor with 2 levels (1 = low, 2 = high)

Each model was run with: options(contrasts = c("contr.sum","contr.poly"))

A1: MM for accuracy & RT data (InputData = whole data set)

```
TM1 <- mixed(Perf ~ 1 + (1|MSUnc) + (1|id), data = InputData, method="KR")
TM2 <- mixed(Perf ~ 1 + TE + (1|MSUnc) + (1|id), data = InputData, method="KR")
TM3 <- mixed(Perf ~ 1 + TE*Mod + (1|MSUnc) + (1|id), data = InputData, method="KR")
TM4 <- mixed(Perf ~ 1 + TE*SpatUnc + (1|MSUnc) + (1|id), data = InputData, method="KR")
TM5 <- mixed(Perf ~ 1 + TE*Know + (1|MSUnc) + (1|id), data = InputData, method="KR")
TM6 <- mixed(Perf ~ 1 + TE*Run + (1|MSUnc) + (1|id), data = InputData, method="KR")
TM7 <- mixed(Perf ~ 1 + TE*Mod*SpatUnc + (1|MSUnc) + (1|id), data = InputData, method="KR")
TM8 <- mixed(Perf ~ 1 + TE*Mod*Know + (1|MSUnc) + (1|id), data = InputData, method="KR")
TM9 <- mixed(Perf ~ 1 + TE*Mod*Run + (1|MSUnc) + (1|id), data = InputData, method="KR")
TM10 <- mixed(Perf ~ 1 + TE*SpatUnc*Know + (1|MSUnc) + (1|id), data = InputData, method="KR")
TM11 <- mixed(Perf ~ 1 + TE*SpatUnc*Run + (1|MSUnc) + (1|id), data = InputData, method="KR")
TM12 <- mixed(Perf ~ 1 + TE*Know*Run + (1|MSUnc) + (1|id), data = InputData, method="KR")
TM13 <- mixed(Perf ~ 1 + TE*Mod*SpatUnc*Know + (1|MSUnc) + (1|id), data = InputData, method="KR")
TM14 <- mixed(Perf ~ 1 + TE*Mod*SpatUnc*Run + (1|MSUnc) + (1|id), data = InputData, method="KR")
TM15 <- mixed(Perf ~ 1 + TE*Mod*Know*Run + (1|MSUnc) + (1|id), data = InputData, method="KR")
TM16 <- mixed(Perf ~ 1 + TE*Mod*SpatUnc*Run*Know + (1|MSUnc) + (1|id), data = InputData, method="KR")
```

A2: MM for accuracy & RT data (InputData = low OR high modality-spec. uncertainty)

```
TM1 <- mixed(Perf ~ 1 + (1|id), data = InputData, method="KR")
TM2 <- mixed(Perf ~ 1 + TE + (1|id), data = InputData, method="KR")
TM3 <- mixed(Perf ~ 1 + TE*Mod + (1|id), data = InputData, method="KR")
TM4 <- mixed(Perf ~ 1 + TE*SpatUnc + (1|id), data = InputData, method="KR")
TM5 <- mixed(Perf ~ 1 + TE*Know + (1|id), data = InputData, method="KR")
TM6 <- mixed(Perf ~ 1 + TE*Run + (1|id), data = InputData, method="KR")
TM7 <- mixed(Perf ~ 1 + TE*Mod*SpatUnc + (1|id), data = InputData, method="KR")
TM8 <- mixed(Perf ~ 1 + TE*Mod*Know + (1|id), data = InputData, method="KR")
TM9 <- mixed(Perf ~ 1 + TE*Mod*Run + (1|id), data = InputData, method="KR")
TM10 <- mixed(Perf ~ 1 + TE*SpatUnc*Know + (1|id), data = InputData, method="KR")
TM11 <- mixed(Perf ~ 1 + TE*SpatUnc*Run + (1|id), data = InputData, method="KR")
TM12 <- mixed(Perf ~ 1 + TE*Know*Run + (1|id), data = InputData, method="KR")
TM13 <- mixed(Perf ~ 1 + TE*Mod*SpatUnc*Know + (1|id), data = InputData, method="KR")
TM14 <- mixed(Perf ~ 1 + TE*Mod*SpatUnc*Run + (1|id), data = InputData, method="KR")
TM15 <- mixed(Perf ~ 1 + TE*Mod*Know*Run + (1|id), data = InputData, method="KR")
TM16 <- mixed(Perf ~ 1 + TE*Mod*SpatUnc*Run*Know + (1|id), data = InputData, method="KR")
```

B1: MM for learn trial data (InputData = whole data set)

```
LTM1  <- mixed(Perf ~ 1 + (1|MSUnc) + (1|id), data = InputData, method="KR")
LTM2  <- mixed(Perf ~ 1 + Mod + (1|MSUnc) + (1|id), data = InputData, method="KR")
LTM3  <- mixed(Perf ~ 1 + Know + (1|MSUnc) + (1|id), data = InputData, method="KR")
LTM4 <- mixed(Perf ~ 1 + SpatUnc + (1|MSUnc) + (1|id), data = InputData, method="KR")
LTM5  <- mixed(Perf ~ 1 + Mod*Know + (1|MSUnc) + (1|id), data = InputData, method="KR")
LTM6  <- mixed(Perf ~ 1 + Mod*SpatUnc + (1|MSUnc) + (1|id), data = InputData, method="KR")
LTM7  <- mixed(Perf ~ 1 + SpatUnc*Know + (1|MSUnc) + (1|id), data = InputData, method="KR")
LTM8  <- mixed(Perf ~ 1 + Mod*SpatUnc*Know + (1|MSUnc) + (1|id), data = InputData, method="KR")
```

B2: MM for learn trial data (InputData = low OR high modality-spec. uncertainty)

```
LTM1  <- mixed(Perf ~ 1 + (1|id), data = InputData, method="KR")
LTM2  <- mixed(Perf ~ 1 + Mod + (1|id), data = InputData, method="KR")
LTM3  <- mixed(Perf ~ 1 + Know + (1|id), data = InputData, method="KR")
LTM4 <- mixed(Perf ~ 1 + SpatUnc + (1|id), data = InputData, method="KR")
LTM5  <- mixed(Perf ~ 1 + Mod*Know + (1|id), data = InputData, method="KR")
LTM6  <- mixed(Perf ~ 1 + Mod*SpatUnc + (1|id), data = InputData, method="KR")
LTM7  <- mixed(Perf ~ 1 + SpatUnc*Know + (1|id), data = InputData, method="KR")
LTM8  <- mixed(Perf ~ 1 + Mod*SpatUnc*Know + (1|id), data = InputData, method="KR")
```
